# Gene Expression Analysis of Induced *Plum pox virus* (Sharka) Resistance in Peach (*Prunus persica*) by Almond (*P. dulcis*) Grafting

**DOI:** 10.3390/ijms22073585

**Published:** 2021-03-30

**Authors:** Manuel Rubio, Pedro J. Martínez-García, Azam Nikbakht-Dehkordi, Ángela S. Prudencio, Eva M. Gómez, Bernardo Rodamilans, Federico Dicenta, Juan A. García, Pedro Martínez-Gómez

**Affiliations:** 1 Department of Plant Breeding, CEBAS-CSIC, P.O. Box 164, 30100 Murcia, Spain; mrubio@cebas.csic.es (M.R.); pjmartinez@cebas.csic.es (P.J.M.-G.); asanchez@cebas.csic.es (Á.S.P.); eva.gomezgonzalez1@bayer.com (E.M.G.); fdicenta@cebas.csic.es (F.D.); 2 Department of Plant Breeding and Biotechnology, Faculty of Crop Sciences, Sari Agricultural Sciences and Natural Resources University, P.O. Box 578, Sari, Iran; azam.nikbakht.dehkordi@gmail.com; 3 Department of Plant Molecular Genetics, CNB-CSIC, 28049 Madrid, Spain; brodamilans@cnb.csic.es (B.R.); jagarcia@cnb.csic.es (J.A.G.)

**Keywords:** PPV, sharka disease, peach, almond, breeding, plant–virus interaction, RNA-Seq, RTqPCR

## Abstract

No natural sources of resistance to *Plum pox virus* (PPV, sharka disease) have been identified in peach. However, previous studies have demonstrated that grafting a “Garrigues” almond scion onto “GF305” peach rootstock seedlings heavily infected with PPV can progressively reduce disease symptoms and virus accumulation. Furthermore, grafting a “Garrigues” scion onto the “GF305” rootstock has been shown to completely prevent virus infection. This study aims to analyse the rewiring of gene expression associated with this resistance to PPV transmitted by grafting through the phloem using RNA-Seq and RT-qPCR analysis. A total of 18 candidate genes were differentially expressed after grafting “Garrigues” almond onto healthy “GF305” peach. Among the up-regulated genes, a *HEN1* homolog stands out, which, together with the differential expression of *RDR*- and *DCL2*-homologs, suggests that the RNA silencing machinery is activated by PPV infection and can contribute to the resistance induced by “Garrigues” almond. *Glucan endo-1,3-beta D-glucosidase* could be also relevant for the “Garrigues”-induced response, since its expression is much higher in “Garrigues” than in “GF305”. We also discuss the potential relevance of the following in PPV infection and “Garrigues”-induced resistance: several pathogenesis-related proteins; no apical meristem proteins; the transcription initiation factor, TFIIB; the speckle-type POZ protein; in addition to a number of proteins involved in phytohormone signalling.

## 1. Introduction

Stone (*Prunus*) fruit trees are affected by a large number of viral diseases that can cause important economic losses [1]. However, Sharka, caused by *Plum pox virus* (PPV), is the most significant of these diseases due to the reduced fruit quality and premature fruit drop that it causes [2]. This pathogen has been classified as a quarantine pathogen and as one of the Top 10 Viruses in crops [3,4]. Currently, however, the best current method for controlling PPV is preventing the spread of the virus to new fruit-growing areas [5], although genetic resistance is the definitive control strategy for PPV in affected areas [1,6,7]. However, in the case of peach (*P. persica* (L.) Batsch)—the most important *Prunus* species, with an annual production of 25.7 million tons in 2019 (http://www.fao.org/faostat/en/#data, accessed on 24 February 2021)—no natural sources of resistance have been identified [8,9]. The lack of resistance to PPV in peach could be solved by incorporating resistant genes from other related species, such as *P. ferganensis* (Kostov and Rjabov) and *P. davidiana* (Carrière) Franch. Nevertheless, the transmission of resistance from these related species has been questioned, and the importance of the genetic background in the effectiveness of this strategy has been highlighted [10]. Another alternative for introducing PPV resistance into peach, is the use of the closely related almond (*P. dulcis* (Miller) Webb) species [11].

Previous studies have demonstrated that grafting the “Garrigues” almond scion onto “GF305” peach rootstock (a very susceptible PPV indicator) seedlings heavily infected with PPV can progressively reduce disease symptoms and virus accumulation [7]. This response appears to be specific between almond and peach. Furthermore, grafting the “Garrigues” scion onto the “GF305” rootstock before PPV inoculation completely prevented virus infection, showing that resistance is constitutive and not induced by the virus [12]. In a recent study, results showed that early PPV infection in leaves without disease symptoms is associated with the induction of genes coding for factors involved in pathogen resistance, such as jasmonate biosynthesis elements, chitinases and Lys-M proteins. On the other hand, when the virus was installed, the overexpression of dicer-like (DCL) protein 2a genes could demonstrate the induction of defensive responses related to RNA silencing, counteracted by the HCPro (helper component proteinase) silencing suppressor of the virus [13]. In addition, a recent comparison of hormonal balances in healthy and PPV-inoculated “GF305” peach rootstocks, either grafted or not grafted with the almond cultivar “Garrigues”, showed important differences between treatments [14]. PPV inoculation produced a significant increase in gibberellin GA3 and abscisic acid (ABA) and a decrease in the other phytohormones analysed, including the cytokinin trans-zeatine (tZ), gibberellin GA4, ethylene precursor 1-aminocyclopropane-1-carboxylic acid (ACC), salicylic acid (SA) and jasmonic acid (JA). Additionally, grafting “Garrigues” onto “GF305” produced an increase in GA3, GA4, SA and ABA and a decrease in the rest of the phytohormones analysed (tZ, ACC and JA). However, grafting “Garrigues” almond onto PPV-inoculated “GF305” peach produced the opposite effect in some phytohormones, resulting in an increase in tZ, SA and JA. The results of this work suggest a major role of SA in almond-induced resistance in peach, with additional contributions from tZ and JA.

On the other hand, it is well known that viruses induce different defense responses related to RNA silencing in different plant species [15,16]. However, the PPV resistance produced by “Garrigues” grafting-induced systemic resistance (ISR) in peach does not require an initial viral infection, suggesting that this response is not linked with the induction of antiviral RNA silencing, nor is it related to responses such as ISR or systemic acquired resistance (SAR) [17]. It, therefore, seems more likely that an unknown specific factor of “Garrigues” almond spreads through the graft to “GF305” and contributes to blocking the multiplication or movement of the virus. In this context, the mobility of various macromolecules including DNA, RNA and proteins has been well documented between the scion and stock, suggesting that the graft could be a means of horizontal genetic transfer [18,19]. Not just phenotypic traits, but also the core molecular building blocks could be altered in the grafted individuals [20,21]. In addition, endogenous small RNAs of all size classes (21–24 nt) can move across the graft union [22]. This movement can give rise to specific physiological reprogramming and epigenetic changes in different tissues [23,24].

Our work aims to analyze the differential gene expression linked to the resistance to PPV transmitted to peach “GF305” via grafting with “Garrigues” almond cultivars, focusing on the differential expression patterns shown and comparing the different treatments studied.

## 2. Results

### 2.1. PPV Resistance Evaluation

**Figure 1:**
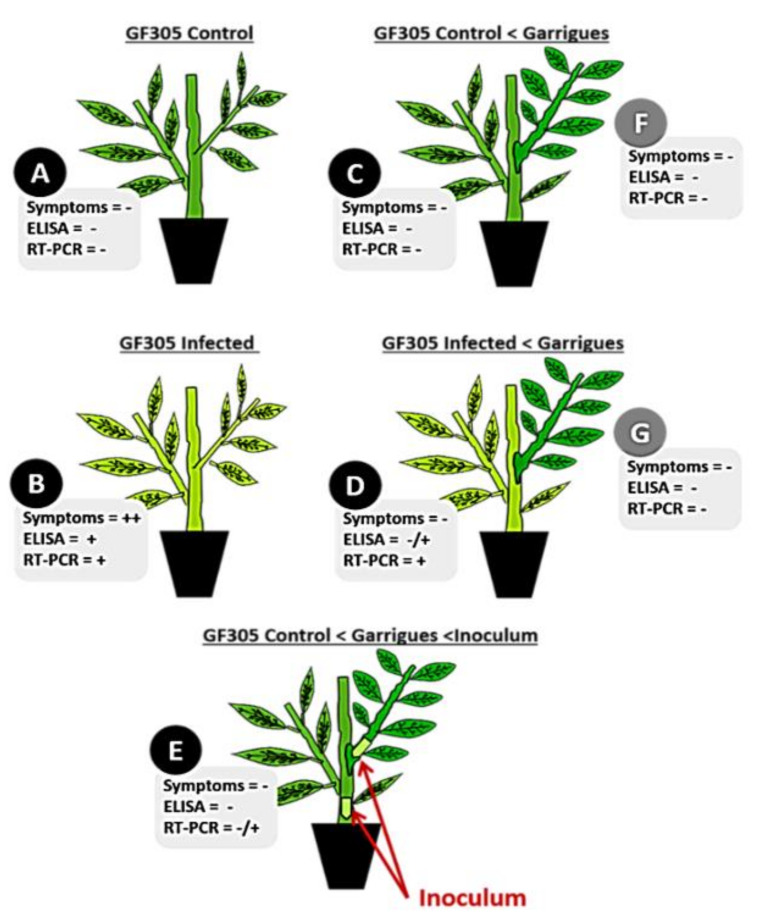
Schematic representation of the assayed treatments including symptoms, ELISA and RT-PCR: (**A**) control healthy “GF305” peach rootstocks; (**B**) *Plum pox virus* (PPV)-infected “GF305” rootstocks showing severe sharka symptoms; (**C**) healthy “GF305” peach rootstocks grafted with “Garrigues”; (**D**) PPV-infected “GF305” rootstocks showing sharka symptoms grafted with “Garrigues”; (**E**) healthy “GF305” peach rootstocks grafted with “Garrigues”, which were later inoculated with PPV at the end of the first cycle of evaluation. In addition, “Garrigues” almond samples grafted onto healthy (**F**) and PPV-inoculated “GF305” (**G**) rootstocks were included in the study. PPV was evaluated by symptom observation in leaf, ELISA-test and RT-PCR analysis. Presence of virus using these methodologies was indicated with + and absence with -.

The results shown in Figure 1 and Table 1 confirm the high susceptibility of “GF305” peach to PPV (all inoculated plants showed clear symptoms and were ELISA-DASI and RT-PCR positive in sample B) and the resistance of the almond variety “Garrigues” to the assayed PPV isolate (no plants showed disease symptoms or were ELISA-DASI or RT-PCR positive (samples F and G)). In the first two phenotyping cycles, four infected “GF305” plants grafted with “Garrigues” (D) were positive by ELISA and RT-PCR, and only one of them showed noticeable symptoms during the second cycle. In addition, we performed a third phenotyping cycle, during which, one of the infected “GF305” plants grafted with “Garrigues” still showed the presence of PPV but was symptomless. The effect of “Garrigues” grafting was also very clear when “GF305” plants already grafted with “Garrigues” were inoculated (sample E). In this case, we never observed symptoms on the “GF305” rootstock, and only one plant gave positive RT-PCR results.

### 2.2. RNA-Seq Transcriptome Profiles

Electropherograms of RNA samples for RNA-Seq analysis have been added as Supplementary File S1. A total of 1024 million paired end reads of 125 bp (base pair) were generated from the seven samples tested (Table 2). After eliminating adapter sequences, empty reads and low-quality sequences, 1021 million high-quality reads (99%), which were named clean reads, were obtained. By iterative alignment, 75.8% of the clean reads were mapped in the reference genome Peach v2.0 (www.rosaceae.org; accessed on 21 January 2021), while 24.2% of the clean reads were not recognised by any of the eight pseudo region-chromosomes. Alignment values in the peach genome ranged from an average of 78.7% in the peach samples to 68.2% in the almond samples. On the other hand, 2.15 million unmapped reads (0.21% of the total clean reads) were mapped in the PPV genome (Table 2). We can assume that 15, 152 and 44 supposed PPV reads from control plants (samples A, C and F, respectively) are contaminations. Ultimately, we were able to observe the clear effect of grafting “Garrigues” almond onto “GF305” peach. Comparing sample B with sample D, we noticed a 25-fold decrease in PPV-mapped reads during the second phenotyping cycle. Additionally, in the case in which “GF305” was grafted with “Garrigues” and then inoculated with PPV (sample E), the number of reads was almost 1000 times lower than in infected non-grafted “GF305” (B) (Table 2). These data fit well with symptom observation and virus detection data obtained from ELISA and RT-PCR during the second cycle of evaluation in which samples were collected (Table 1). “Garrigues” sample G showed 45 PPV-mapped reads. Although we cannot rule out the possibility that these few reads were derived from virus that remained in the infected “GF305” rootstock after the alleviation due to almond grafting, they are probably contaminations similar those found in samples A, C and F.

### 2.3. Host Transcriptional Changes

Table 3 summarises the differences in gene expression between the different samples analysed. The total number of expressed peach genes that were tested in the different comparisons ranged from 18,832 in the D/C comparison, to 20,643 in the D/B comparison. A total of 18,808 expressed genes were tested in the comparison of almond samples (G/F). In addition, 5815 differently expressed genes (DEGs) were identified in the different comparisons analysed using the cuffmerge and cuffdiff tool (Table 3). Considering the effect of PPV inoculation/infection, 1102 and 548 DEGs were detected on “GF305” after inoculation (comparison B/A, Supplementary Table S1) and inoculation plus almond grafting (comparison D/C, Supplementary Table S2), respectively. Moreover, 439 DEGs were detected on “Garrigues” almond when PPV-inoculated and non-inoculated plants were compared (comparison G/F; Supplementary Table S3). In assessing the effect of grafting “Garrigues” almond onto healthy “GF305”, 823 DEGs were detected (comparison C/A; Supplementary Table S4). Focusing on both effects in “GF305” (PPV infection + “Garrigues” grafting), we studied two comparisons, D/B (Supplementary Table S5) and E/B (Supplementary Table S6), which showed 1163 and 1113 DEGs, respectively. The last comparison performed was to study the effect of shifting the order of inoculation and “Garrigues” grafting (comparison E/D, Supplementary Table S7) with 627 DEGs. After filtering the data by log-fold change > 1 and <−1) and q-val < 0.05, the number of DEGs decreased drastically to 197 genes (147 unique) (Table 3). No differentially expressed genes were detected between “Garrigues” grafted onto infected or healthy rootstocks (G/F). As regards the effect of PPV infection/inoculation on “GF305”, 44 genes were detected in non-grafted plants (B/A), while in comparisons between grafted and non-grafted plants D/B and E/B, 31 and 47 filtered DEGs were detected, respectively, with 14 shared genes. Comparing the infected “GF305” grafted with “Garrigues” (D) with the same treatment but shifting the order of inoculation and grafting (E/D) and with control “GF305” grafted with “Garrigues” (D/C), we observed a similar number of filtered DEGs (28 and 29, respectively), with eight shared genes in this case (Table 3). The effect of almond grafting on healthy “GF305” rootstocks (comparison C/A) showed the lowest number of filtered DEGs (18), with an equilibrium between up- and down-regulation (10/8). We observed that the heavy infection of sample B (infected “GF305” control) generally triggered the largest deregulation of genes.

### 2.4. Functional Analysis of Differentially Expressed Genes

**Figure 2:**
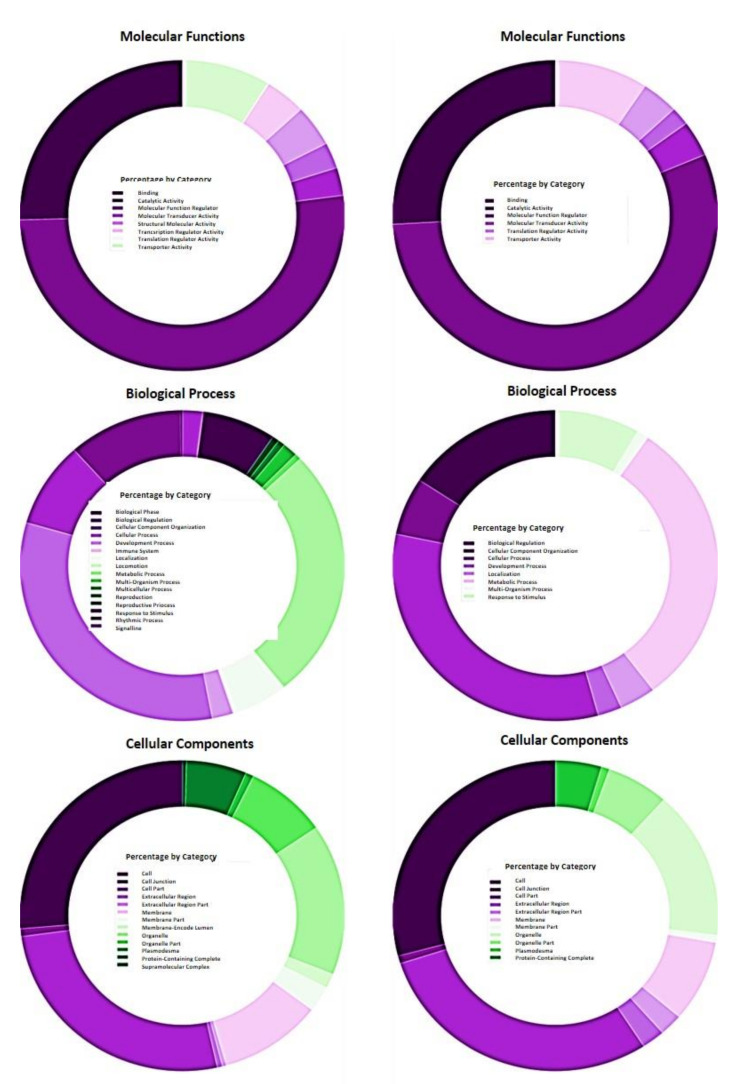
GO term categories obtained by PANTHER. Functional analysis of differentially expressed genes including all comparisons (3210; left side) and functional analysis of filtered differentially expressed genes (147; right side). Diagrams show percentage of GO term categories showing the total of each domain for “Cellular Components”, “Biological Processes” and “Molecular Functions” domains.

**Figure 3:**
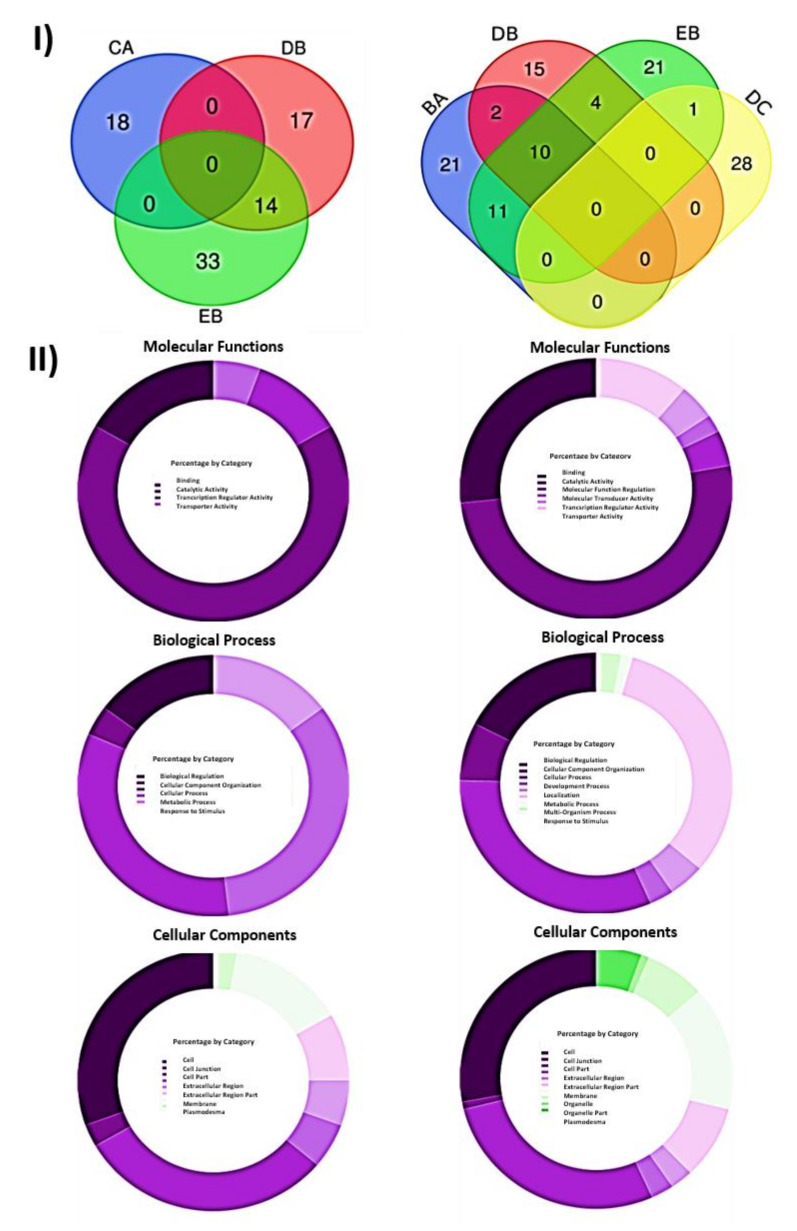
(**I**) Analysis of DEGs associated with grafting (CA vs. DB vs. EB) (left) and inoculation (BA vs. DB vs. EB vs. DC) (right) effects: control healthy “GF305” peach rootstocks; PPV-infected “GF305” rootstocks showing severe sharka symptoms; “GF305” peach rootstocks grafted with healthy “Garrigues”; PPV-infected “GF305” rootstocks showing sharka symptoms grafted with healthy “Garrigues”; healthy “GF305” peach rootstock grafted with healthy “Garrigues” which were later inoculated with PPV at the end of the first cycle of evaluation. “Garrigues” almond samples grafted onto healthy and inoculated “GF305” rootstocks. (**II**) GO term categories obtained by PANTHER for DEGs associated with grafting (left) and inoculation (right) effects. Diagram shows percentage of GO term categories showing the total of each domain for DEGs “Cellular Components”, “Biological Processes” and “Molecular Functions” domains.

The 5815 differentially expressed genes (DEGs) across samples represented a total of 3269 different genes. From this set, 3210 were assigned with a UNIPROTKB ID, and 1115 were assigned with one or more GO terms using PANTHER (Supplementary Table S8; Figure 2). The most represented functional category was “Cellular Component” with 2782 hits, followed by “Biological Process” and “Molecular Function”, with 1721 and 1058 hits, respectively. The most represented cellular component terms were cell (GO:0005623) and cell part (GO:0044464), with 713 hits each, followed by the “Biological Process” term cellular process (GO:0009987), with 558 hits. Another highly represented “Cellular Component” term was organelle (GO:0043226) (438 hits). The term with the second highest percentage of hits (453 hits) in “Biological Process” was metabolic process (GO:0008152). As regards “Molecular Function” terms, the most represented term was catalytic activity (546 hits). The 197 filtered DEGs (Table 3) corresponded to 147 different genes. Only one gene could not be mapped by PANTHER (Prupe_2G011500), and 56 were associated with at least one GO term (Supplementary Table S9; Figure 2). The distribution pattern of GO terms in this restricted list was similar to that of the complete set of DEGs (Figure 3). Again, the functional category with the highest number of hits was “Cellular Component” with 130 hits, followed by “Biological Process” and “Molecular Function” with 83 and 54 hits, respectively. As in the total set of DEGs, the most represented terms were two “Cellular Component” terms, cell (GO:0005623) and cell part (GO:0044464), with 38 hits each. The most represented “Biological Process” terms were cellular process (GO:0009987) and metabolic process (GO:0008152), with 27 and 25 hits, respectively. For “Molecular Function”, the most represented term was catalytic activity (GO:0003824) (30 hits).

The overrepresentation analysis was carried out with PANTHER to compare our complete DEG list with the reference peach genome (Supplementary Table S8). In the case of “Biological Process”, the results showed the highest over-representation for several GO terms, such as posttranscriptional gene silencing (GO:0016441), posttranscriptional gene silencing by RNA (GO:0035194) and gene silencing by RNA (GO:0031047) (5.19-fold enrichment). Other terms with remarkable over-representation were beta-glucan biosynthetic process (GO:0051274), mitotic cytokinesis (GO:0000281) and cytoskeleton-dependent cytokinesis (GO:0061640). Most of the GO terms of the DEGs in our experiment with statistically significant different representation compared to the peach genome were under-represented. Under representation was maximum for the terms tRNA metabolic process (GO:0006399) and peptidyl-amino acid modification (GO:0018193) (impoverishment values of 5.58- and 4.17-fold, respectively. As regards “Cellular Component”, the only terms significantly over-represented were cytosolic large ribosomal subunit (GO:0022625), cytosolic ribosome (GO:0022626) and cytosolic part (GO:0044445), with fold enrichments of 2.09, 1.96, and 1.73, respectively. Again, the number of under-represented terms in our DEG list is larger than that of over-represented terms, and membrane protein complex (GO:0098796) was the term showing the highest under-representation (three-fold). Only five “Molecular Function” terms showed a significantly different representation in our DEG list compared to the peach genome (four enriched terms and one impoverished). In this case, calcium ion binding (GO:0005509) was the most prominent term, with a 3.14-fold enrichment value. No statistically significant results were obtained in the overrepresentation analysis with PANTHER of the filtered DEG set for “Molecular Function” and “Cellular Component” terms (Supplementary Table S9). No significantly under-represented “Biological Process” terms were found either. However, 11 “Biological Process” terms showed a significant over-representation in the filtered DEG set. The three brother terms, posttranscriptional gene silencing (GO:0016441), posttranscriptional gene silencing by RNA (GO:0035194) and gene silencing by RNA (GO:0031047), with a 56.64-fold enrichment value, were already identified in the total DEG set. Interestingly, the rest of the over-represented GO terms in the restricted DEG set were also related to the silencing and regulation of gene expression.

Next, the GO analysis was refined by grouping together the filtered DEGs associated with common biological effects: control healthy “GF305” peach rootstocks (A); PPV-infected “GF305” rootstocks showing severe sharka symptoms (B); “GF305” peach rootstocks grafted with healthy “Garrigues” (C); PPV-infected “GF305” rootstocks showing sharka symptoms grafted with healthy “Garrigues” (D); healthy “GF305” peach rootstock grafted with healthy “Garrigues” which were later inoculated with PPV at the end of the first cycle of evaluation (E). “Garrigues” almond samples grafted onto healthy (F) and inoculated “GF305” (G) rootstocks. Comparisons associated with the almond grafting effect (CA vs. DB vs. EB, Table 3) yielded a total of 82 filtered DEGs, none of which were shared by the three comparisons (Figure 3I and Supplementary Table S10). The comparisons of conditions joining almond grafting and PPV infection (DB vs. EB, Table 3) shared a total of 14 filtered DEGs (Figure 3I and Supplementary Table S10). In this set of 82 filtered DEGs, only one could not be mapped by PANTHER, and the number of genes annotated with at least one GO term was 19. The functional category with the highest number of hits was “Cellular Component” with 36 hits, followed by “Biological Process” and “Molecular Function” with 27 and 18 hits, respectively. The most represented term was the “Molecular Function” term catalytic activity (GO:0003824) (12 hits), followed by two “Cellular Component” terms, cell (GO:0005623) and cell part (GO:0044464) (11 hits each), and by two “Biological Process” terms, metabolic process (GO:0008152) and cellular process (GO:0009987) (nine hits each) (Supplementary Table S10 and Figure 3). No statistically significant over- or under-represented terms were found in the overrepresentation analysis for this set of filtered DEGs (Supplementary Table S10).

The four comparisons associated with PPV inoculation/infection (BA vs. DB vs. EB vs. DC, Table 3) resulted in 113 filtered DEGs, none of which were shared by the four comparisons (Figure 3I and Supplementary Table S11). Comparisons of heavily infected non-grafted “GF305” vs. “GF305” with low or no virus load, either non-grafted (BA) or grafted (DB and EB), shared 10 filtered DEGs (Figure 3I and Supplementary Table S11). In addition, one filtered DEG was shared between the comparisons of almond-grafted and non-grafted PPV-inoculated trees (EB) and PPV-inoculated and non-inoculated almond-grafted “GF305” (DC). In this set of 113 DEGs related to PPV infection, 42 DEGs were annotated with at least one GO-SLIM term, with 225 hits (Supplementary Table S11). Again, the functional category with the highest number of hits was “Cellular Component” with 111 hits, followed by “Biological Process” and “Molecular Function” with 69 and 45 hits, respectively. Among the “Cellular Component” terms, cell (GO:0005623) and cell part (GO:0044464) showed the highest number of hits (31), followed by catalytic activity (GO:0003824) (23 hits) in the “Molecular Function” category, and cellular process (GO:0009987) (22 hits) and metabolic process (GO:0008152) (22 hits) in the “Biological Process” domain. Thirteen GO terms assigned by PANTHER corresponding to the “Biological Process” category were significantly overrepresented in this subset of filtered DEGs in comparison with the *P. persica* reference list (Supplementary Table S11). Nine of these were also overrepresented in the complete list of filtered DEGs, including the three most enriched terms (posttranscriptional gene silencing, GO:0016441; posttranscriptional gene silencing by RNA, GO:0035194; and gene silencing by RNA, GO:0031047) (Supplementary Tables S9 and S11). The enrichment analysis did not obtain significant results for the “Molecular Function” or “Cellular Component” categories (Supplementary Table S11).

### 2.5. Validation of Gene Expression Profiles Using RT-qPCR

**Figure 4:**
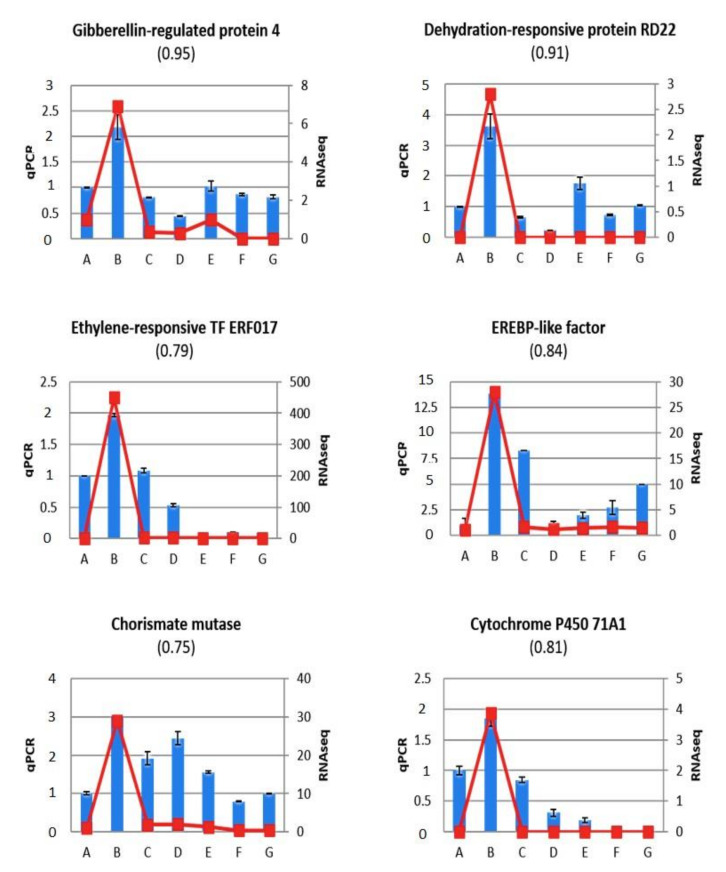
RT-qPCR expression analysis (blue bars) for candidate genes involved in phytohormonal signalling, including a phytohormone related to growth, such as gibberellin (GA) (Prupe.5G193400; *gibberellin-regulated protein 4*); phytohormones related to stress, such as abscisic acid (ABA) (Prupe.1G453700; *dehydration-responsive protein RD22*); ethylene (Prupe.7G194400; *ethylene-responsive transcription factor ERF017* and Prupe.4G176200; *ethylene responsive element binding protein* (*ERBP*)-*like factor*); salicylic acid (SA) (Prupe.1G393400; *chrorismate mutase*; (Prupe.5G164200; *cytochrome P450 71A1* (*CYP71A1*), all selected from the RNA-Seq analysis. Relative gene expression (RGE) in the seven samples: control healthy “GF305” peach rootstocks (A); PPV-infected “GF305” rootstocks showing severe sharka symptoms (B); “GF305” peach rootstocks grafted with healthy “Garrigues” (C); PPV-infected “GF305” rootstocks showing sharka symptoms grafted with healthy “Garrigues” (D); healthy “GF305” peach rootstock grafted with healthy “Garrigues” which were later inoculated with PPV at the end of the first cycle of evaluation (E). “Garrigues” almond samples grafted onto healthy (F) and inoculated “GF305” (G) rootstocks. Error bars in qPCR represent the standard error of three independent biological replicates. Between brackets: Pearson correlation coefficients between FPKM (fragments per kilobase pair of transcript per million mapped reads) values from RNA-Seq (in red lines) and RGE values from qPCR.

**Figure 5:**
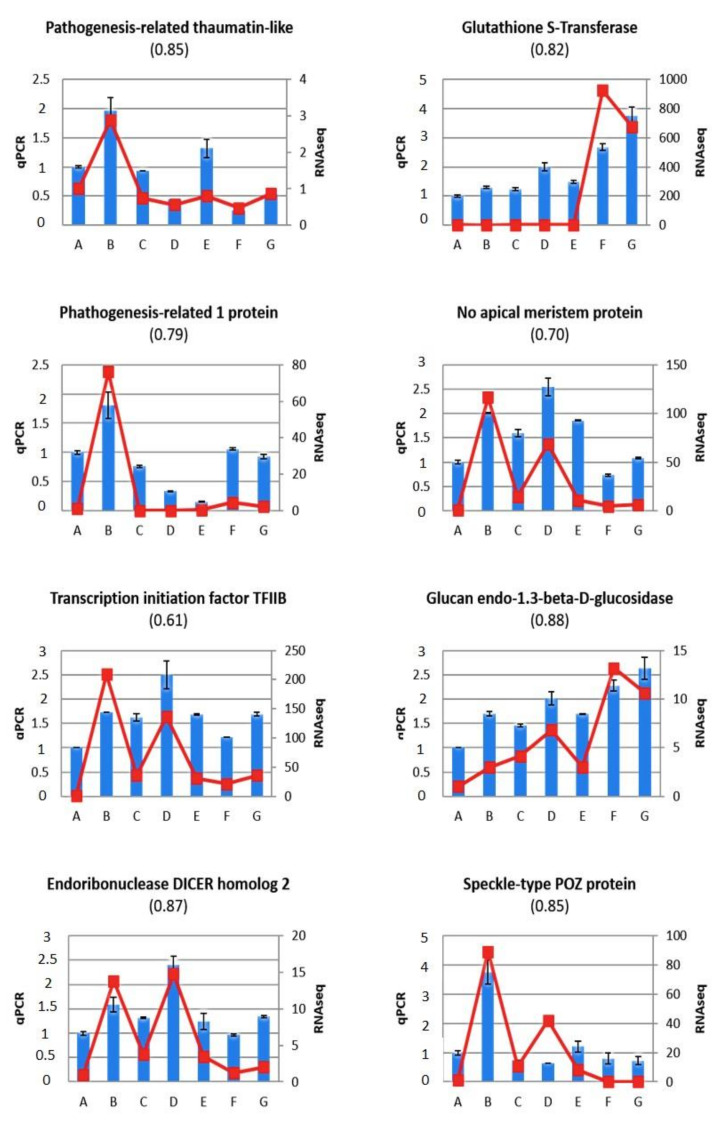
RT-qPCR expression analysis for candidate genes related to the pathogen response selected from the RNA-Seq analysis (blue bars) including: *Pathogenesis-related thaumatin-like protein* (Prupe.8G163300), *glutathione S-transferase* (Prupe.1G039900), *pathogenesis-related 1 protein* (Prupe.1G091400), *no apical meristem protein* (Prupe.2G204700), *transcription initiation factor TFIIB* (Prupe.3G255800), *glucan endo-1,3-beta-D-glucosidase* (Prupe.7G051200), *endoribonuclease DICER homolog 2* (*DCL2*) (Prupe.7G048000) and *speckle-type POZ protein* (Prupe.1G488200). Relative gene expression (RGE) in the seven samples: control healthy “GF305” peach rootstocks (A); PPV-infected “GF305” rootstocks showing severe sharka symptoms (B); “GF305” peach rootstocks grafted with healthy “Garrigues” (C); PPV-infected “GF305” rootstocks showing sharka symptoms grafted with healthy “Garrigues” (D); healthy “GF305” peach rootstock grafted with healthy “Garrigues” which were later inoculated with PPV at the end of the first cycle of evaluation (E). “Garrigues” almond samples grafted onto healthy (F) and inoculated “GF305” (G) rootstocks. Error bars in qPCR data represent the standard error of three independent biological replicates. Between brackets: Pearson correlation coefficients between FPKM (fragments per kilobase pair of transcript per million mapped reads) values from RNA-Seq (in red lines) and RGE values from qPCR.

Fourteen genes were selected based on different criteria for validating RNA-Seq data by RT-qPCR analyses in an additional experiment (Figure 4 and Figure 5). A melting curve analysis of the expression profiles of these genes is included in the Supplementary Materials as Supplementary File S2. Some of these genes were selected for their link with the signalling of phytohormones, including a hormone related to gibberellin (GA) growth (Prupe.5G193400; gibberellin-regulated protein 4) and phytohormones related to stress, such as abscisic acid (ABA) (Prupe.1G453700; dehydration-responsive protein RD22); ethylene (Prupe.7G194400; ethylene-responsive transcription factor ERF017 and Prupe.4G176200; ethylene responsive element binding protein (ERBP)-like factor); salicylic acid (SA) (Prupe.1G393400; chrorismate mutase and Prupe.5G164200; cytochrome P450 71A1 (CYP71A1).

Most of selected candidate genes, however, code for factors related to the pathogen response, such as a pathogenesis-related thaumatin-like protein (Prupe.8G163300), glutathione S-t*ransferase* (Prupe.1G039900), a p*athogenesis-related 1 protein* (Prupe.1G091400), a *no apical meristem protein* (Prupe.2G204700), transcription initiation factor TFIIB (Prupe.3G255800), glucan endo-1,3-beta-D-glucosidase (Prupe.7G051200) and the endoribonuclease DICER homolog 2 (DCL2) (Prupe.7G048000). The differential expression of the gene encoding the s*peckle-type POZ protein* (Prupe.1G488200) is of special interest, since this protein has a meprin and TRAF homology (MATH) domain, and proteins with this domain have been found to be involved in resistance to PPV. The expression trends of eight of the selected genes were similar to those shown by RNA-Seq analysis. Pearson correlation coefficients between fragments per kilobase pair of transcript per million mapped reads (FPKM) values from RNA-Seq and relative gene expression (RGE) values from RT-qPCR were higher than 0.70 for 12 of the 14 assayed genes. *Transcription initiation factor-TFIIB* and no apical meristem protein (*NAM*) are exceptions, with correlation coefficients of 0.61 and 0.70, respectively. The anomalous values of speckle-type POZ protein in sample D represent another example of the low correlation between the two analyses. The expression of most analysed genes was higher in leaves showing disease symptoms (sample B) than in non-symptomatic leaves. In addition, grafting “Garrigues” induced an increase in the expression of the following genes: *EREBP-like factor*, *chorismate mutase, no-apical meristem protein*, *transcription initiation factor-TFIIB*, *lucan endo-1.3-beta-D-glucosidase* and *DCL2*.

## 3. Discussion

The “protection effect” of the “Garrigues” scion was clear in the “GF305” rootstock that was inoculated when “Garrigues” had already been grafted (sample E). In this case, no symptoms were ever observed on “GF305”. From the agronomical point of view, this is the most important effect, since we can graft “Garrigues” as an intermediate wood on commercial rootstocks (interstock), and later, when the scions have developed, we can graft the peach varieties. The specific combination that gives resistance to PPV should be evaluated in other peach varieties. These results also open the possibility of using “Garrigues” as a rootstock or interstock to control PPV in peach, similar to the proposed solution in grapevine [25] and tomato [26]. The intermediate rootstock model is widely used in agriculture to solve compatibility problems between cultivars and species, or to induce specific behaviours such as dwarfing, reduced juvenility, etc. [27]. In these studies, it has been well evidenced that genetic “factors”, such as small RNAs, can travel from scions to the rootstocks in order to control—in an epigenetic manner—the rootstock’s genes, with this movement being carried out by the phloem. However, the opposite has been shown in vegetables, with this movement in the opposite direction being slower. Given that we are interested in the production of high-quality peach fruits produced by the scions, it must be proven that the “Garrigues” almond cultivar can have the same effect as a rootstock as it has as a scion.

The PPV-resistance response induced by grafting “Garrigues” onto “GF305” is associated with a strong transcriptomic imbalance with many changes at the mRNA level, as previously described for grafting in other plant species [20,25,26]. The total number of filtered DEGs detected in our seven comparisons combining grafting and infection effects was relatively low (197) compared to previous studies after PPV-infection in peach, with 1554 DEGs in three comparisons [13], or apricot (*P. armeniaca* L.), with 437 DEGs in only two comparisons [28]. This low number is probably related to improvements in the phenotyping and sampling method, i.e., selecting all leaves with a similar age, development stage and phytosanitary status in order to reduce effects not linked to PPV or grafting. It is conceivable that a signal from “Garrigues” moves through the graft to “GF305” and elicits the resetting program that confers protection against PPV. If we assume that this induction of “resistance” is independent of PPV presence, we must focus on the C/A comparison (Supplementary Table S4). Only 18 DEGs were detected in this comparison. Among these genes, 10 genes were up-regulated after grafting healthy “GF305” with “Garrigues” almond (sample C). Notably, the level of expression of these 10 genes was also higher in “GF305” plants that were inoculated with PPV after grafting with “Garrigues” and were not infected (sample E), compared to healthy non-grafted “GF305” (sample A) (Supplementary Tables S4 and S6). These up-regulated genes can therefore be considered as potential candidates for involvement in the defensive response induced by “Garrigues”.

One of the DEGs of the C/A comparison that attracts the most attention is Prupe.4G091400, which is identified as the peach homolog of HUA ENHANCER 1 (HEN1). This DEG encodes an RNA methyltransferase that plays an important role in RNA silencing by methylating siRNAs [29]. This gene was associated with the brother GO-SLIM terms posttranscriptional gene silencing (GO:0016441), posttranscriptional gene silencing by RNA (GO:0035194) and gene silencing by RNA (GO:0031047). HEN1 was highly enriched in our complete list of DEGs and filtered DEGs (Supplementary Tables S8 and S9) as well as in the list of filtered DEGs related to PPV infection (Supplementary Table S11). These GO terms were also associated with the genes Prupe.1G132200, Prupe.4G078900, Prupe.1G332600 and Prupe.1G334500, which code for putative RNA-dependent RNA polymerases (RDR) (Supplementary Table S9). Although differences were not shown to be statistically significant in the C/A comparison, these four genes were expressed at higher levels in non-infected “GF305” grafted with “Garrigues” (sample C) than in the healthy non-grafted “GF305” control (sample A) (Supplementary Table S4). An RNA silencing-related gene that has been reported to be activated by PPV infection in woody plants is DCL2 [13,28]. Confirming the relationship of DCL2 with PPV infection, this gene (Prupe.7G048000) was significantly overexpressed in “GF305” trees that recovered from PPV infection by “Garrigues” grafting (sample D) compared to grafted “GF305” trees that never suffered PPV infection (samples C and E) (Supplementary Table S12). This differential expression was confirmed by RT-qPCR analysis. As in the case of the aforementioned RDR genes, the expression of Prupe.7G048000 was higher in non-infected “GF305” grafted with “Garrigues” (sample C) than in uninfected “GF305” (sample A), although in this case, differences did not reach the threshold of statistical significance (Supplementary Table S4 and Figure 5).

A recent translatome (entirety actively translated mRNAs) profiling study identified one HEN1 homolog, three DCL2 homologs, six RDR1 homologs, and other predicted RNA silencing-related genes that were up-regulated in both the phloem and non-phloem tissues of plum leaves developing PPV infection after a period of cold-induced dormancy [30]. Activation in the phloem tissue is especially interesting because it could provide a mobile defensive barrier for crossing the grafting junction. These data agree with the results of our study and suggest that antiviral defenses related to RNA silencing contribute to the virus resistance established in the “GF305” rootstock after grafting with the “Garrigues” scion almond. Another interesting gene showing statistically significant differential expression in the C/A comparison is Prupe.7G051200, which was identified as a glucan endo-1,3-beta D-glucosidase coding for a ß-1,3-glucanase of the thaumatin family of pathogenesis-related (PR) proteins. It has been reported that virus infection can induce a distinct ß-1,3-glucanase activity in mutant plants that are deficient in this enzymatic activity [31]. Moreover, a thaumatin-like protein accumulated in the apoplast of infected peach after PPV infection [32]. In agreement with these findings, the RNA-seq analysis showed an increase (not statically significant) in the expression of Prupe.7G051200 as well as of Prupe.8G163300 and Prupe.1G091400, encoding another PR-related thaumatin-like protein and a PR 1-like protein, respectively, associated with PPV infection in non-grafted “GF305” plants (B/A comparison, Supplementary Table S12). This was confirmed by RT-qPCR (Figure 5). More importantly, “Garrigues” almond (samples G and F) showed a much higher level of expression of Prupe.7G051200 than “GF305” peach (sample A), as revealed by RNA-seq (Supplementary Tables S1 and S3) and RT-qPCR (Figure 5), and grafting of “Garrigues” almond caused a significant increase in the Prupe.7G051200 activity of “GF305” peach (Supplementary Table S12 and Figure 5). The relevance of this increase in the “Garrigues”-induced resistance is unclear, since deficiency in ß-1,3-glucanase activity has been shown to decrease, rather than enhance, susceptibility to viral disease [31]. Both grafting and infection effects may contribute to the differences in the expression of the 14 DEGs shared in the comparisons DB and EB (Supplementary Table S10). However, the absence of significant expression differences between samples C and A in all these genes, and the fact that the infection of non-grafted “GF305” plants caused significant changes in the expression (mainly up-regulation) of most of them (Supplementary Table S11), suggests that the effect of infection exceeds that of grafting in these DB and EB comparisons.

An important contribution of phytohormonal signalling in PPV infection and resistance has been revealed through a metabolomic analysis conducted by Nikbakht-Dehkordi et al. [14]. In agreement with this observation, at least four genes differentially expressed in the BA, BD and BE comparisons were involved in phytohormone signalling. These genes, Prupe.1G393400, Prupe.4G176200, Prupe.5G164200 and Prupe.1G453700, were identified as *Chorismate mutase*, *EREBP-like factor*, *CYP71A1*, and *Dehydration-responsive RD22* (Supplementary Table S12), and their high expression levels in infected non-grafted “GF305” (sample B) were confirmed by RT-qPCR (Figure 4). SA is well known to be involved in plant defence mechanisms, and this hormone has been shown to be involved in the decrease in symptoms induced by grafting “Garrigues” onto PPV-infected “GF305” peach [14]. In this regard, chorismate mutase is a key enzyme in the biosynthetic pathway of SA [33], and the cytochrome P450 CYP71A1, which encodes the tryptamine 5-hydroxylase that catalyses the conversion of tryptamine to serotonin, is involved in the cross-talk between salicylic acid and serotonin biosynthetic pathways controlling plant defence responses [34]. There are numerous reports showing the relevance of ABA in plant virus infection. In particular, ABA has recently been shown to play a negative role in the response to PPV infection [35]. Dehydration-responsive RD22 is a classical marker of an activated ABA response [36], and its overexpression in the B sample (Supplementary Table S12 and Figure 4) is consistent with the important role played by ABA in PPV infection. The main role of the phytohormone ethylene in plant resistance, is in the defense response to necrotrophic pathogens, but there are also reports showing its involvement in viral infections [37]. The involvement of ethylene in PPV is soundly supported by the strong induction of an EREBP-like factor gene associated with a symptomatic PPV infection (sample B, Supplementary Table S12, Figure 4). This assumption was reinforced by the much higher expression level of the DEG Prupe.7G194400, identified as ethylene-responsive transcription factor ERF017, in infected “GF305” compared to the healthy control (247.05 vs. 0.559, Supplementary Table S1). Although it could not be considered statistically significant, this difference in expression level was confirmed by an RT-qPCR assay (Figure 4).

To explore the possible involvement of other phytohormones in “GF305” infection and its recovery by grafting with “Garrigues”, we analysed a phytohormone-related gene that did not appear to be differentially expressed in the RNA-seq experiment via RT-qPCR. Prupe.5G193400, identified as giberellin-regulated protein 4, showed consistent data in the RNA-seq and RT-qPCR analyses, revealing a notable over-accumulation in the PPV-infected non-grafted RT-qPCR analyses. This result suggests that gibberellins also play a role in the PPV infection of peach trees, and, together with the results discussed above, reflects the complex pattern of phytohormone rearrangements associated with the PPV infection of “GF305” peach and the resistance induced by “Garrigues” almond, as previously discussed by Nikbakht-Dehkordi et al. [14]. Prupe.2G204700 and Prupe.3G255800 were identified as *no apical meristem protein* (NAM) *and transcription initiation factor* (*TFIIB*), respectively. NAM proteins are included within the NAC family, a family including members involved in interactions with pathogens [38]. These proteins are transcription factors that can act as negative regulators of resistance to pathogens by suppressing defence-related gene expression. Some NAC proteins can enhance or inhibit the multiplication of viruses by interacting directly with virus-encoded proteins [39,40]. *TFIIB* is a general transcription factor that forms part of the pre-initiation complex of RNA polymerase II. The structure of *TFIIB* presents two domains, an imperfect repeat of approximately 70 aa and a *TFII* zinc-binding domain. Proteins with this last domain have been shown to be involved in the regulation of responses to biotic and abiotic stresses and in resistance to pathogens [41]. Both RNA-seq and RT-qPCR data revealed induction of Prupe.2G204700 and Prupe.3G255800 by PPV infection (compare B and A samples in Figure 5). Of note, the expression of these two genes was hardly reduced when PPV infection was mitigated by “Garrigues” grafting, and was enhanced when “Garrigues” was grafted onto healthy “GF305” trees (compare samples D vs. B and C vs. A in Figure 5). These results suggest that NAM and TFIIB could be relevant not only for PPV infection but also for its healing via “Garrigues” grafting.

Prupe.1G488200 was identified as a speckle-type POZ protein, a gene encoding an E3 ubiquitin ligase holding a meprin and TRAF homology (MATH) domain [42]. In *Arabidopsis thaliana* the MATH-TRAF gene, RTM3, encodes a factor involved in the restriction of systemic spread of different potyviruses, including PPV [43], and at least one other PPV resistance gene has been mapped to a region encompassing seven MATH-TRAF genes [44]. More importantly, PPV resistance in apricot cultivars has been extensively linked to the repression of ParPMC1 and ParPMC2 genes, both encoding MATH family proteins, which are located in the major resistance locus PPVres [45,46]. The downregulation of these genes has been suggested to be caused by an RNA silencing mechanism triggered by the pseudogenisation of the resistance allele of *ParPMC2* [46,47]. In our study, none of the *MATH-TRAF* genes of the peach syntenic region of the apricot PPVres locus were differentially expressed. However, the expression of Prupe.1G488200, which is also located in chromosome 1, but downstream of the *PPVres*-related region, was induced by PPV infection (sample B), showing lower expression levels in healthy and grafting-induced recovered peaches (Figure 5). Whether the role of speckle-type POZ protein in peach infection is related to that of *ParPMC1* and *ParPMC2* in apricot and RTM3-like proteins in *Arabidopsis* remains unknown. This study identified candidate genes involved in peach infection by PPV and recovery induced by “Garrigues” almond grafting. However, a larger number of biological replicas is required to enhance the statistical significance of the results. In addition, detailed analyses assessing the intermediate time points in the process of viral recovery would provide stronger conclusions about the factors involved in the resistance induced by “Garrigues” grafting. On the other hand, we must not forget that changes in the efficiency of RNA translation or in post-translational modifications of the protein, which would not be detected in RNA-seq experiments, could also contribute to the resistance induced in “GF305” peach. Identifying these factors will require additional experimental approaches.

## 4. Materials and Methods

### 4.1. Biological Materials

Seedlings of peach “GF305” peach are characterised by their susceptibility to fruit viruses including PPV [48] and are used as a rootstock in PPV resistance tests on *Prunus* species under greenhouse conditions [49]. This material was used as rootstock for our grafting experiments with the “Garrigues” almond cultivar. Firstly, “GF305” peach seedlings, healthy or PPV-inoculated and showing sharka symptoms, were analysed. Later we proceeded to the analysis of almond-grafted peach plants (inoculated with PPV and control) to study the phenomenon of induced resistance. In these grafted plants, in order to identify the mechanism of systemic defence against PPV and the effector molecule that is intervening, a differential expression analysis was conducted by high-throughput sequencing mRNA (mRNA-Seq). The mRNA-Seq data were validated by RT-qPCR in different “GF305” and “Garrigues” plants. The PPV isolate used was 3.30RB/GF-IVIA (GenBank: KJ849228.1), a Dideron Type (PPV-D) isolate obtained from the “Red Beaut” Japanese plum variety in Spain in the 1980s and maintained in the PPV collection of the Instituto Valenciano de Investigaciones Agrarias (IVIA) in Valencia (Spain). This isolate produces strong sharka symptoms in young leaves of “GF305” peach, consisting of venial chlorosis and rings.

### 4.2. Sharka Phenotyping and PPV Detection

All the experiments were carried out in a greenhouse under controlled conditions. The complete evaluation procedure was described in Rubio et al. [12]. Briefly, plants were subjected to growth cycles in the greenhouse and rest periods in a cold chamber. Rootstocks were grown, inoculated with PPV and grafted with “Garrigues” almond cultivars. During each evaluation cycle, plants were inspected for sharka symptoms, and PPV was detected by ELISA-DASI (Enzyme-Linked ImmunoSorbent Assay-Double Antibody Sandwich Immunosorbent) and RT-PCR (Reverse Transcription Polimerase Chain Reaction) tests. In the present work, three cycles of phenotyping were performed (Table 1). Sharka symptoms on leaves were scored using a scale from 0 (no symptoms) to 5 (maximum intensity), taking into account intensity and distribution in the plant as follows: 0, no symptoms; 1, discrete chlorosis or spots restricted to one or two leaves; 2, slight chlorosis bordering leaf veins in three or more leaves; 3, vein chlorosis or rings in numerous leaves; 4, chlorosis, rings and some distortions on most leaves; 5, strong chlorosis or distortions on all the leaves. The presence of PPV was confirmed by DASI-ELISA with the specific monoclonal antibody to the capsid protein (CP) of PPV 5B-IVIA/AMR (Plant Print Diagnostics SL, Valencia, Spain). Optical densities (OD) were recorded at 405 nm after 60 min of substrate incubation, and samples with OD double that of the healthy control were considered positive. Finally, for the detection of PPV RNA, total RNA was extracted from the same leaves subjected to the DASI-ELISA test using the RNeasy Plant Mini Kit^®^ (Qiagen, Hilden, Germany), and RT-PCR analysis was carried out using specific primers for the coat protein coding sequence VP337 (5′ CTCTGTGTCCTCTTCTTGTG 3′) and VP338 (5′ CAATAAAGCCATTGTTGGATC 3′). The enzymes used were avian myeloblastosis virus reverse transcriptase and GoTaq^®^Flexi2 polymerase (Promega, Madison, WI). The RT-PCR parameters were 42 °C for 45 min (cDNA synthesis) followed by one step at 94 °C for 2 min, and 35 cycles at 94 °C for 30 s, 55 °C for 30 s and 72 °C for 30 s, with a final extension step at 72 °C for 5 min. RT-PCR amplified products were separated by electrophoresis on 1% agarose gels in 40 mM Tris-acetate and 1mM EDTA, pH 8.0, and stained with GelRed^®^ (Biotium, San Francisco, USA) [49].

### 4.3. Experimental Design and High-Throughput mRNA Sequencing

We assayed the following treatments: control (sample A) and PPV-infected “GF305” peach seedlings showing strong sharka symptoms (sample B); control “GF305” grafted with “Garrigues” almond (samples C and F) and PPV-infected “GF305” grafted with “Garrigues” (samples D and G); control “GF305” grafted with “Garrigues” almond and later inoculated with PPV (sample E) (Figure 1). Leaf samples (a pool of leaves from five plants per replicate) from the evaluation cycle 2 (Table 1) were frozen in liquid nitrogen and stored at −80 °C. Total RNA was extracted using the RNeasy Plant Mini Kit^®^ (Qiagen, Hilden, Germany). The quality and quantity of the total RNA samples were assessed using a NanoDrop^®^ 2000 spectrophotometer (Thermo Fisher Scientific, Wilmington, USA) and diluted at the same concentration (200 ng/µL). RNA samples (5 µg) were sent to the Centre for Genomic Regulation (CRG, Barcelona, Spain) for library preparation and RNA sequencing, assaying 2 cDNA libraries per treatment. The 14 cDNA libraries of poly-A RNA were sequenced using an Illumina HiSeq2000 machine to perform 125 bp paired-end sequencing.

### 4.4. Bioinformatic Analysis

A quality control step was performed for the RNA-Seq (mRNA) reads using PRINSEQ software [50]. In the mRNA analysis, adaptors were trimmed and low-quality bases at the ends of sequences and reads with undetermined bases or with 80% of their bases with less than 20% quality score were removed. High quality mRNA reads were mapped to the reference genome Prunus persica genome v2.0 (http://www.rosaceae.org/peach/genome, accessed on 21 January 2021) [51] using STAR [52]. Genomic annotations were obtained from the Genome Database for Rosaceae (http://www.rosaceae.org/, accessed on 21 January 2021) in general feature format 3 (GFF3). The presence of the PPV virus was quantified by aligning the sequences that did not map to the peach genome against the PPV 3.30RB/GF-IVIA genome sequence (KJ849228.1) (Table 2). The transcript isoform (mRNA) level and gene level counts were calculated and FPKM (fragments per kilobase pair of transcript per million mapped reads) normalised using Cufflinks 2.2.1 software [53]. Total genes as well as tested genes were identified. Some of the total genes identified were not tested because of the very low number of reads detected. Differential transcript expression at the mRNA level was then computed using cuffmerge and cuffdiff [54]. After this, we selected those with a false discovery rate (fdr) <0.05 (*p*-value <0.05). The resulting lists of differentially expressed isoforms (mRNA) were then filtered by log2 (log-fold change) >1 and <−1 and a q-value <0.05, taking into account all the identified genes in each comparison, including up- and down-regulated differentially expressed genes (Table 3, Supplementary Table S12). The total list of unique DEG genes was used to perform a functional classification using PANTHER version 14 [55]. In addition, an overrepresentation test was carried out using a GO-SLIM annotation data set for each functional classification (molecular function, biological process and cellular component), and all genes were listed in the peach genome, according to Fisher’s exact statistical test and Benjamini–Hochberg’s False Discovery Rate correction. Subsequently, results were plotted using an in-house python script using matplotlib [56]. In addition, Venn diagrams were generated using the online tool from the University of Ghent (http://bioinformatics.psb.ugent.be/webtools/Venn, accessed on 21 January 2021).

### 4.5. RT-qPCR Validation of Selected Transcripts

To validate the RNA-Seq analysis, RT-qPCR was performed in a new experiment with new RNA samples from the same initial experiment, assaying three replicates per treatment. Relative quantitative PCR (qPCR) experiments were executed with StepOnePlus™ real-time PCR system (Applied Biosystems). Specific primers were designed based on peach/almond sequences previously obtained using Primer3 software (Supplementary Table S13). A total of 17 qPCR reactions were performed, including 13 differentially expressed new genes and three internal controls, as previously described by Rubio et al. [12]. qPCR efficiency was checked by the standard curve method. For all real-time qPCR reactions, a 10 μL mix was made containing: 5 μL Power SYBR^®^ Green PCR Master Mix (Applied Biosystems), 10 to 20 ng of cDNA, and 2.5 µM of each primer. The qPCR conditions were as follows: 95 °C for 10 min; 40 cycles of 95 °C for 15 s and 60 °C for 1 min. The melting temperature in these experiments was set to 60~95 °C and increased by 0.3 °C/s. Each biological sample was implemented in triplicate. RPII, actin and expansin-A8 were used as reference genes for data normalisation [57], and the levels of relative expression were calculated by the comparative method [58], taking Ct value from the first samples (treatment A) as the reference expression level.

## 5. Conclusions

Again, our results show that resistance to PPV in “Garrigues” can be transmitted through the graft union via the phloem and protect the highly susceptible “GF305” peach. We propose that grafting provides a path for horizontal gene transfer. Although we cannot determine the specific mechanisms of resistance that regulate this surprising behavior, several candidate “resistance” genes have been identified, including defense genes such as *RTM* genes, *NAM proteins*, *TFIIB*, *TLPs* (thaumatin like protein), and *dehydration-responsive proteins*. These candidate “resistance” genes were over-expressed after “Garrigues” grafting. In addition, several candidate genes (including *glucan endo-1,3-beta D-glucosidase*, *leucine rich repeat N-terminal domain*, and *speckle-type POZ protein*) up-regulated after almond grafting should be considered as “susceptible” genes that are necessary for PPV infection. This down-regulation of the susceptible gene candidates could be due to a gene methylation process mediated by an sRNA molecule. Based on these results, we can deduce the following resistant hypothesis for grafting “Garrigues” to peach. First, grafting induced a series of signal transduction genes at high expression levels, with an interesting correlation among constitutive thaumatin-like protein, PR proteins, ABA compound, and disease susceptibility to PPV. Secondly, protein kinase interacts with transcription factors or other factors and up-regulates the expression relevant susceptible genes. On the other hand, if we are ultimately able to corroborate the “Garrigues effect” at the commercial level (nurseries and orchards), we will have an effective strategy against the continuous threat of PPV for peach production.

## Figures and Tables

**Table 1:** Evaluation of behaviour against PPV in the assayed plant material. The samples assayed included control healthy “GF305” peach rootstocks (A); PPV-infected “GF305” rootstocks showing severe sharka symptoms (B); “GF305” peach rootstocks grafted with healthy “Garrigues” (C); PPV-infected “GF305” rootstocks showing sharka symptoms grafted with healthy “Garrigues” (D); healthy “GF305” peach rootstock grafted with healthy “Garrigues” which were later inoculated with PPV at the end of the first cycle of evaluation (E). In addition, “Garrigues” almond samples grafted onto healthy (F) and inoculated “GF305” (G) rootstocks were included in the study. Bold type refers to the evaluated plant material **GF305** peach and **Garrigues** almond.

		Cycle 1			Cycle 2				Cycle 3			
Plant Model	Sample	N ^1^	Symptoms ^2^	ELISA-DASI ^3^	N ^1^	Symptoms ^2^	ELISA-DASI ^3^	RT-PCR ^4^	N ^1^	Symptoms ^2^	ELISA-DASI ^3^	RT-PCR ^4^
**GF305** Control	A	8	0 (0)	0 (0.05)	8	0 (0)	0 (0.05)	0	7	0 (0)	0 (0.06)	0
**GF305** + PPV	B	8	8 (3.1)	8 (3.51)	8	8 (4)	8 (3.12)	8	7	7 (3.1)	7 (1.35)	7
**GF305** Control + Garrigues	C	8	0 (0)	0 (0.05)	7	0 (0)	0 (0.01)	0	6	0 (0)	0 (0.09)	0
**GF305** + PPV + Garrigues	D	8	2 (1)	1 (1.23)	6	1 (1)	5 (1.59)	5	5	0 (0)	1 (0.28)	1
**GF305** Control + Garrigues + PPV	E	8	0 (0)	0 (0.06)	8	0 (0)	0 (0.03)	1	7	0 (0)	0 (0.05)	0
GF305 Control + **Garrigues**	F	8	0 (0)	0 (0.06)	6	0 (0)	0 (0.05)	0	6	0 (0)	0 (0.06)	0
GF305 + PPV + **Garrigues**	G	8	0 (0)	0 (0.05)	7	0 (0)	0 (0.05)	0	5	0 (0)	0 (0.07)	0

^1^ Number of biological replications evaluated; ^2^ Number of repetitions with sharka symptoms, between parenthesis are the mean values of the repetitions with symptoms on a scale from 0 to 5; ^3^ Number of repetitions ELISA-DASI positive, between parenthesis are the mean OD (optical density) values of the assayed replications; ^4^ Number of repetitions RT-PCR positive.

**Table 2:** Mapping characteristics of samples assayed including the two biological replicates by treatment: control healthy “GF305” peach seedlings (A); PPV-infected “GF305” seedlings showing severe sharka symptoms (B); “GF305” peach seedlings grafted with healthy “Garrigues” (clonal) (C); PPV-infected “GF305” seedlings showing sharka symptoms grafted with healthy “Garrigues” (clonal) (D); healthy “GF305” peach seedlings grafted with healthy “Garrigues” (clonal) which were later inoculated with PPV at the end of the first cycle of evaluation (E). In addition, “Garrigues” almond samples grafted onto healthy (F) and inoculated “GF305” (G) rootstocks were included in the study. In bold type: the sequenced plant material, **GF305** = peach; **Garrigues** = almond. With an inverted comma (’) = single forward; double inverted comma (”) = single reverse.

Treatments	Sample	Total Reads	Clean Reads	Reads Mapped *P. persica* v 2.0	Reads Mapped PPV Genome
**GF305 Control**	A (A’+A”)	151,699,038	151,376,821	123,331,659 (81.5%)	15 (0.00001%)
**GF305** **+ PPV**	B (B’+B”)	159,272,116	158,812,159	125,187,942 (78.8%)	2,071,915 (1.30%)
**GF305** **Control + Garrigues**	C (C’+C”)	142,956,238	142,296,978	113,078,192 (79.5%)	152 (0.0001%)
**GF305** **+ PPV + Garrigues**	D (D’+D”)	143,511,454	143,041,550	109,499,620 (76.6%)	80,703 (0.06%)
**GF305** **Control + Garrigues + PPV**	E (E’+E”)	148,551,466	148,233,453	114,097,915 (77.0%)	1904 (0.0013%)
**GF305 Control + Garrigues**	F (F’+F”)	140,514,568	140,158,744	88,206,273 (62.9%)	44 (0.00003%)
**GF305 + PPV + Garrigues**	G (G’+G”)	138,038,412	137,569,619	101,163,774 (73.5%)	45 (0.00003%)

**Table 3:** Total (*p*-value < 0.05) and filtered (q-value < 0.05) differentially expressed genes (DEGs), including the number of up- and down-regulated DEGs in peach and almond genes in the seven comparisons performed comparing control (A) and infected GF305 peach showing strong sharka symptoms (B); control GF305 (C) grafted with Garrigues almond (F) and infected GF305 grafted (D) with Garrigues (G); control GF305 grafted with Garrigues almond and later inoculated with PPV (E). In **bold type**: the evaluated plant material, **GF305** = peach; **Garrigues** = almond.

Comparison of Transcriptomes	Sample Comparison	Total Identified Genes	Total Tested Genes	Total DEG Genes	Filtered DEG Genes	DEG Up	DEG Down
Effect of PPV inoculation/infection							
**GF305** + PPV vs. **GF305** Control	B vs. A	27,121	19,335	1102	44	9	35
**GF305** + PPV + Garrigues vs. **GF305** Control + Garrigues	D vs. C	27,036	18,832	548	29	20	9
GF305 + PPV + **Garrigues** vs. GF305 Control + **Garrigues**	G vs. F	27,203	18,808	439	0	-	-
Effect of almond grafting							
**GF305** Control vs. **GF305** Control + Garrigues	C vs. A	27,011	19,906	823	18	10	8
Effect of PPV infection and almond Grafting							
**GF305** + PPV + Garrigues vs. **GF305** + PPV	D vs. B	27,143	20,643	1163	31	23	8
**GF305** Control + Garrigues + PPV vs. **GF305** + PPV	E vs. B	27,131	19,362	1113	47	30	17
Effect of shifting the order of inoculation and grafting							
**GF305** Control + Garrigues + PPV vs. **GF305** + PPV + Garrigues	E vs. D	26,589	18,865	627	28	6	22
Total				5815	197	98	99

## Data Availability

The datasets generated for this study can be found in the NCBI SRA repository as a Bioproject entitled “Gene expression analysis of induced *Plum pox virus* resistance in peach (*Prunus persica*) by almond (*P. dulcis*) grafting”, with the accession number PRJNA712938 (https://www.ncbi.nlm.nih.gov/bioproject/712938, accessed on 21 January 2021).

## Supplementary Materials

The following are available online at https://www.mdpi.com/article/10.3390/ijms22073585/s1, File S1: Electropherograms of RNA samples for RNA-Seq analysis, File S2: Melting Curve Analysis of the expression profiles the assayed genes through RT-qPCR, Table S1: Total genes identified, total genes tested; total (*p*-value < 0.05) and filtered (q-value < 0.05) differentially expressed (DE) genes in the B/A comparison (GF305 + PPV vs. GF305 Control). In bold type: the evaluated plant material, GF305 = peach, Table S2: Total genes identified, total genes tested; total (*p*-value < 0.05) and filtered (q-value < 0.05) differentially expressed (DE) genes in the D/C comparison (GF305 + PPV + Garrigues vs. GF305 Control + Garrigues). In bold type: the evaluated plant material, GF305 = peach and Garrigues = almond, Table S3: Total genes identified, total genes tested; total (*p*-value < 0.05) and filtered (q-value < 0.05) differentially expressed (DE) genes in the G/F comparison (GF305 + PPV + Garrigues vs. GF305 Control + Garrigues). In bold type: the evaluated plant material, GF305 = peach and Garrigues = almond, Table S4: Total genes identified, total genes tested; total (*p*-value < 0.05) and filtered (q-value < 0.05) differentially expressed (DE) genes in the C/A comparison (GF305 Control + Garrigues vs. GF305 Control). In bold type: the evaluated plant material, GF305 = peach and Garrigues = almond, Table S5: Total genes identified, total genes tested; total (*p*-value < 0.05) and filtered (q-value < 0.05) differentially expressed (DE) genes in the D/B comparison (GF305 + PPV + Garrigues vs. GF305 + PPV). In bold type: the evaluated plant material, GF305 = peach and Garrigues = almond, Table S6: Total genes identified, total genes tested; total (*p*-value < 0.05) and filtered (q-value < 0.05) differentially expressed (DE) genes in the E/B comparison (GF305 Control + Garrigues + PPV vs. GF305 + PPV). In bold type: the evaluated plant material, GF305 = peach and Garrigues = almond, Table S7: Total genes identified, total genes tested; total (*p*-value < 0.05) and filtered (q-value < 0.05) differentially expressed (DE) genes in the E/D comparison (GF305 + PPV + Garrigues vs. GF305 Control + Garrigues + PPV). In bold type: the evaluated plant material, GF305 = peach and Garrigues = almond, Table S8: Total list of unique DEG genes (3210), including all comparisons, used to perform an overrepresentation test by Fisher’s exact statistical test and Benjamini–Hochberg’s False Discovery Rate, Table S9: Total list of unique filtered DEG genes (147) including all comparisons, used to perform an overrepresentation test by Fisher’s exact statistical test and Benjamini–Hochberg’s False Discovery Rate, Table S10: Total list of unique filtered DEG genes (82) including comparisons CA vs. DB vs. EB used to perform an overrepresentation test by Fisher’s exact statistical test and Benjamini–Hochberg’s False Discovery Rate, Table S11: Total list of unique filtered DEG genes (113) including comparisons BA vs. DB vs. EB vs. DC used to perform an overrepresentation test by Fisher’s exact statistical test and Benjamini–Hochberg’s False Discovery Rate, Table S12: Total filtered DEG comparisons with up- and down-regulated genes, including comparisons B/A; C/A; D/B; E/B; D/C and E/D. Additionally, we included five genes that do not pass the DEG filter, Table S13: Primer sequences representing a set of sixteen differentially expressed genes in the different treatments and three internal controls selected for qPCR.

## Abbreviations

CYP71A1	Cytochrome P450 71A1
DCL	Dicer-like
DEG	Differently expressed gene
ELISA-DASI	Enzyme-linked immunosorbent assay-double antibody sandwich mmunosorbent
GA	Gibberellic Acid
FPKM	Fragments per kilobase pair of transcript per million mapped reads
GO	Gene ontology
HCPro	Helper component proteinase
HEN1	HUA ENHANCER 1
JA	Jasmonic acid
ISR	Induced systemic resistance
MATH	Meprin and TRAF homology
OD	Optical density
PPV	*Plum pox virus*
PR	Pathogenesis-related
RDR	RNA-dependent RNA polymerase
RNA	Ribonucleic acid
RGE	Relative gene expression
RT-PCR	Reverse transcription polymerase chain reaction
SA	Salicylic acid
SAR	Systemic acquired resistance
TF	Transcription factor
tZ	Trans-zeatine
