# Supplementary material for: Gene Expression Analysis of Induced Plum pox virus (Sharka) Resistance in Peach (Prunus persica) by Almond (P. dulcis) Grafting
Source: Int J Mol Sci. 2021 Mar 30;22(7):3585. doi: 10.3390/ijms22073585 (PMC8036523; doi:10.3390/ijms22073585)
Supplement: Supplementary file 1 [file ijms-22-03585-s001.zip › ijms-1159338 R1 Supplementary Materials/ijms-1159338 R1 File S1.pdf]

Assay Class:	Eukaryote Total RNA Nano	Created:	5/6/2015 9:36:22 AM
Data Path:	\\1...Eukaryote Total RNA Nano_DE23101893_2015-05-06_09-36-22.xad	Modified:	5/6/2015 3:58:01 PM

Electrophoresis File Run Summary

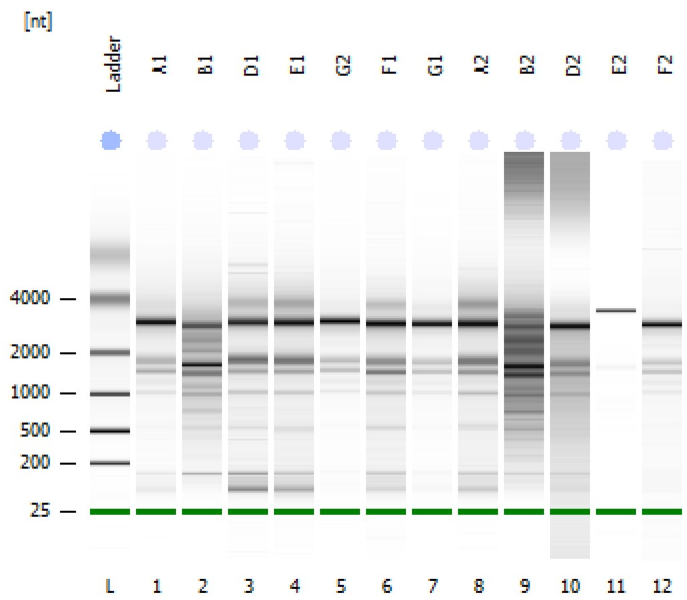

Instrument Information:

Instrument Name:	DE23101893	Firmware:	C.01.069
Serial#:	DE23101893	Type:	G2938B

Assay Information:

Assay Origin Path:	C:\Program Files\Agilent\2100 bioanalyzer\2100 expert\assays\RNA\Eukaryote Total RNA Nano Series II.xsy
Assay Class:	Eukaryote Total RNA Nano
Version:	2.6
Assay Comments:	Total RNA Analysis ng sensitivity (Eukaryote)

© Copyright 2003 - 2009 Agilent Technologies, Inc.

Chip Information:

Chip Lot #:

Reagent Kit Lot #:

Chip Comments:

A1

RIN: 7.80

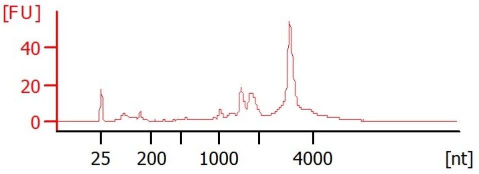

B1

RIN: 5.30

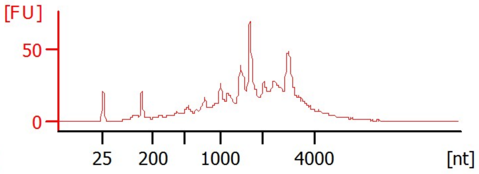

D1

RIN: 7.30

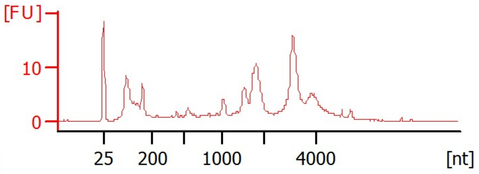

E1

RIN: 7.10

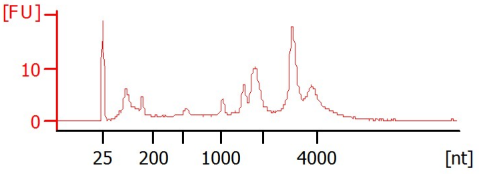

G2

RIN: 8.30

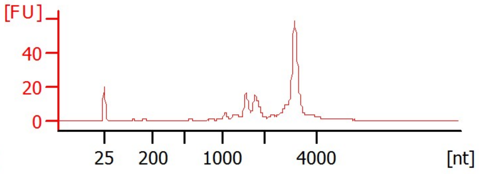

F1

RIN: 7.20

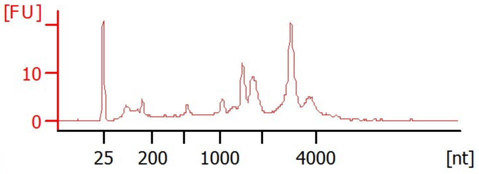

G1

RIN: 8.20

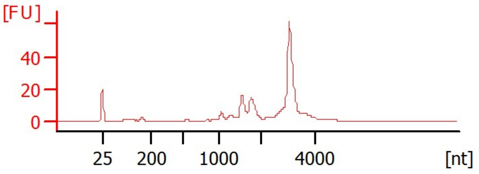

A2

RIN:7

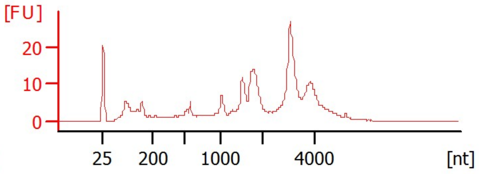

B2

RIN: 3.30

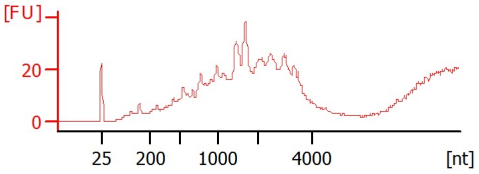

D2

RIN: 6.10

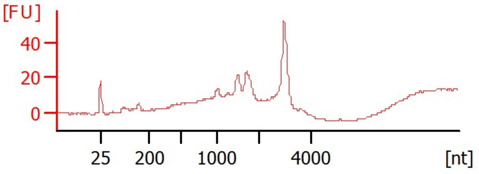

E2

RIN: 6.90

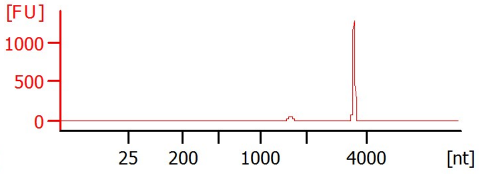

F2

RIN: 8.30

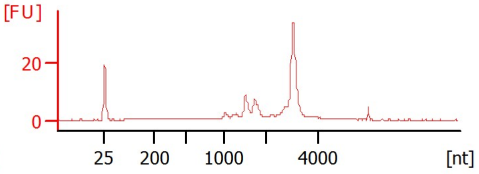

Assay Class: Eukaryote Total RNA Nano
Data Path: \\1...Eukaryote Total RNA Nano_DE23101893_2015-05-06_09-36-22.xad
Created: 5/6/2015 9:36:22 AM
Modified: 5/6/2015 3:58:01 PM

Electrophoresis File Run Summary (Chip Summary)

Sample Name	Sample Comment	Status	Result Label	Result Color
A1		✔	RIN: 7.80	
B1		✔	RIN: 5.30	
D1		✔	RIN: 7.30	
E1		✔	RIN: 7.10	
G2		✔	RIN: 8.30	
F1		✔	RIN: 7.20	
G1		✔	RIN: 8.20	
A2		✔	RIN:7	
B2		✔	RIN: 3.30	
D2		✔	RIN: 6.10	
E2		✔	RIN: 6.90	
F2		✔	RIN: 8.30	
Ladder		✔	All Other Samples	

Chip Lot # **Reagent Kit Lot #**

Chip Comments :

 © Copyright 2003 - 2009 Agilent Technologies, Inc.

Assay Class: Eukaryote Total RNA Nano
Data Path: \\1...Eukaryote Total RNA Nano_DE23101893_2015-05-06_09-36-22.xad
Created: 5/6/2015 9:36:22 AM
Modified: 5/6/2015 3:58:01 PM

Electropherogram Summary

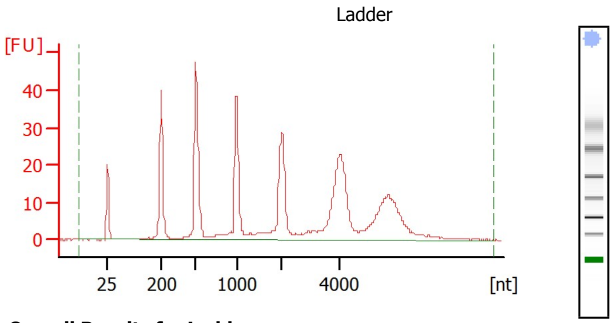

Overall Results for Ladder

RNA Area:	472.6
RNA Concentration:	150 ng/µl
Result Flagging Color:	
Result Flagging Label:	All Other Samples

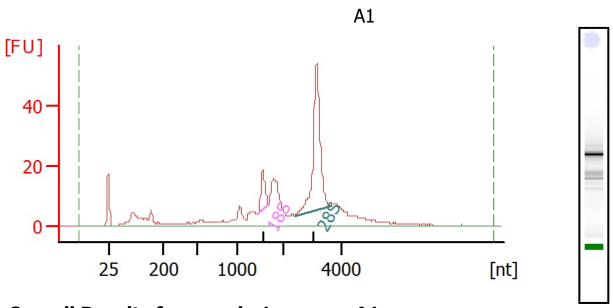

Overall Results for sample 1 : A1

RNA Area:	386.8
RNA Concentration:	123 ng/µl
rRNA Ratio [28s / 18s]:	5.5
RNA Integrity Number (RIN):	7.8 (B.02.08, Anomaly Threshold(s) manually adapted)
Result Flagging Color:	
Result Flagging Label:	RIN: 7.80

Fragment table for sample 1 : A1

Name	Start Size [nt]	End Size [nt]	Area	% of total Area
18S	1,435	1,672	17.4	4.5
28S	2,389	3,621	96.2	24.9

© Copyright 2003 - 2009 Agilent Technologies, Inc.

Assay Class: Eukaryote Total RNA Nano
Data Path: \\1...Eukaryote Total RNA Nano_DE23101893_2015-05-06_09-36-22.xad
Created: 5/6/2015 9:36:22 AM
Modified: 5/6/2015 3:58:01 PM

Electropherogram Summary Continued ...

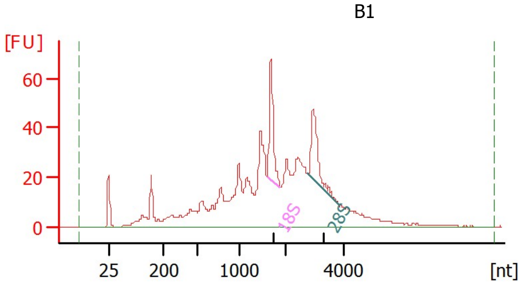

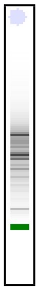

Overall Results for sample 2 : B1

RNA Area:	977.5
RNA Concentration:	310 ng/µl
rRNA Ratio [28s / 18s]:	0.9
RNA Integrity Number (RIN):	5.3 (B.02.08)
Result Flagging Color:	
Result Flagging Label:	RIN: 5.30

Fragment table for sample 2 : B1

Name	Start Size [nt]	End Size [nt]	Area	% of total Area
18S	1,610	1,902	59.5	6.1
28S	2,790	3,847	55.9	5.7

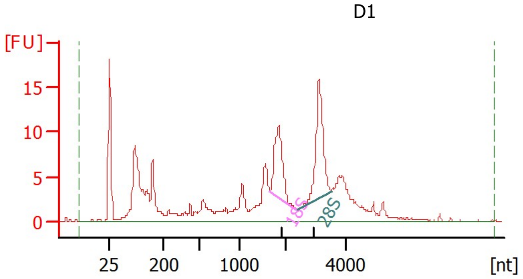

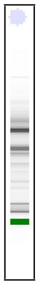

Overall Results for sample 3 : D1

RNA Area:	228.8
RNA Concentration:	73 ng/µl
rRNA Ratio [28s / 18s]:	1.4
RNA Integrity Number (RIN):	7.3 (B.02.08)
Result Flagging Color:	
Result Flagging Label:	RIN: 7.30

Fragment table for sample 3 : D1

Name	Start Size [nt]	End Size [nt]	Area	% of total Area
18S	1,650	2,305	20.2	8.8
28S	2,390	3,543	27.8	12.2

 © Copyright 2003 - 2009 Agilent Technologies, Inc.

Assay Class: Eukaryote Total RNA Nano
Data Path: \\1...Eukaryote Total RNA Nano_DE23101893_2015-05-06_09-36-22.xad
Created: 5/6/2015 9:36:22 AM
Modified: 5/6/2015 3:58:01 PM

Electropherogram Summary Continued ...

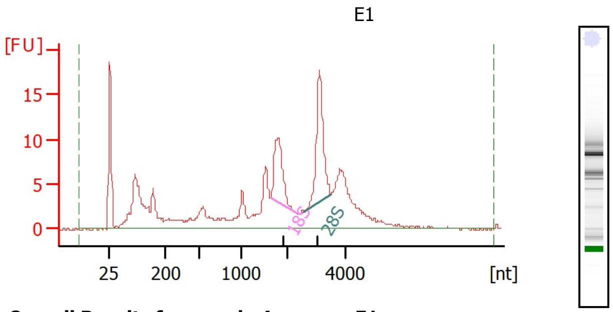

Overall Results for sample 4 : E1

RNA Area:	225.2
RNA Concentration:	71 ng/µl
rRNA Ratio [28s / 18s]:	1.5
RNA Integrity Number (RIN):	7.1 (B.02.08)
Result Flagging Color:	
Result Flagging Label:	RIN: 7.10

Fragment table for sample 4 : E1

Name	Start Size [nt]	End Size [nt]	Area	% of total Area
18S	1,651	2,263	19.6	8.7
28S	2,590	3,484	29.8	13.2

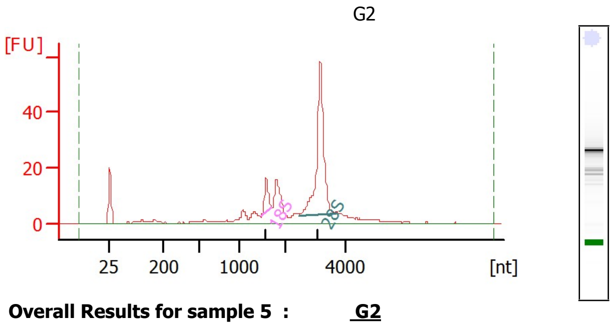

Overall Results for sample 5 : G2

RNA Area:	298.4
RNA Concentration:	95 ng/µl
rRNA Ratio [28s / 18s]:	6.9
RNA Integrity Number (RIN):	8.3 (B.02.08, Anomaly Threshold(s) manually adapted)
Result Flagging Color:	
Result Flagging Label:	RIN: 8.30

Fragment table for sample 5 : G2

Name	Start Size [nt]	End Size [nt]	Area	% of total Area
18S	1,459	1,695	15.8	5.3
28S	2,447	3,643	108.3	36.3

© Copyright 2003 - 2009 Agilent Technologies, Inc.

Assay Class:	Eukaryote Total RNA Nano	Created:	5/6/2015 9:36:22 AM
Data Path:	\\1...Eukaryote Total RNA Nano_DE23101893_2015-05-06_09-36-22.xad	Modified:	5/6/2015 3:58:01 PM

Electropherogram Summary Continued ...

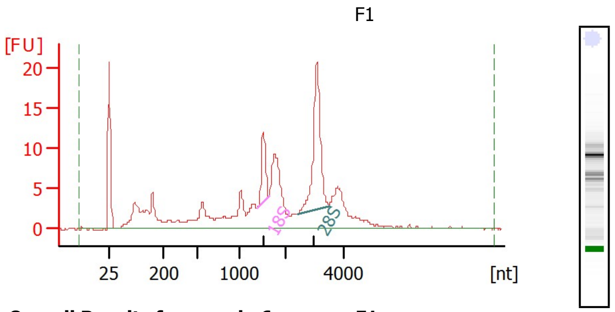

Overall Results for sample 6 : F1

RNA Area:	222.5
RNA Concentration:	71 ng/µl
rRNA Ratio [28s / 18s]:	3.2
RNA Integrity Number (RIN):	7.2 (B.02.08)
Result Flagging Color:	
Result Flagging Label:	RIN: 7.20

Fragment table for sample 6 : F1

Name	Start Size [nt]	End Size [nt]	Area	% of total Area
18S	1,393	1,639	11.7	5.3
28S	2,418	3,488	37.0	16.6

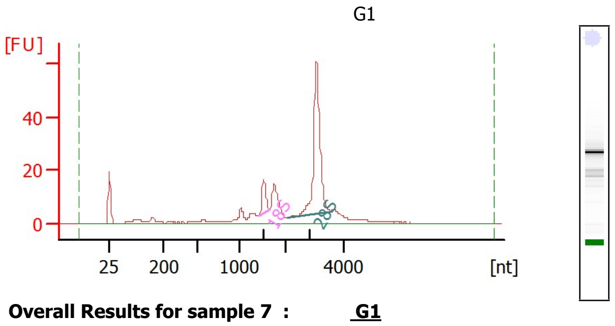

Overall Results for sample 7 : G1

RNA Area:	303.4
RNA Concentration:	96 ng/µl
rRNA Ratio [28s / 18s]:	6.3
RNA Integrity Number (RIN):	8.2 (B.02.08, Anomaly Threshold(s) manually adapted)
Result Flagging Color:	
Result Flagging Label:	RIN: 8.20

Fragment table for sample 7 : G1

Name	Start Size [nt]	End Size [nt]	Area	% of total Area
18S	1,430	1,657	16.4	5.4
28S	2,076	3,545	103.7	34.2

 © Copyright 2003 - 2009 Agilent Technologies, Inc.

Assay Class: Eukaryote Total RNA Nano
Data Path: \\1...Eukaryote Total RNA Nano_DE23101893_2015-05-06_09-36-22.xad
Created: 5/6/2015 9:36:22 AM
Modified: 5/6/2015 3:58:01 PM

Electropherogram Summary Continued ...

A2

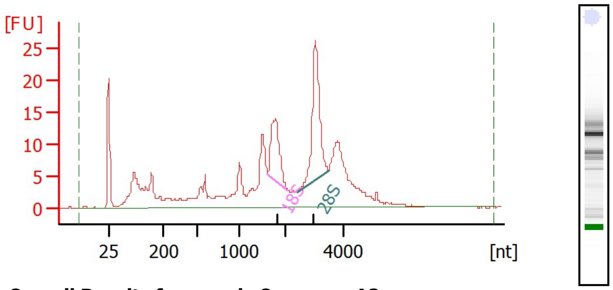

Overall Results for sample 8 : A2

RNA Area:	329.9
RNA Concentration:	105 ng/µl
rRNA Ratio [28s / 18s]:	1.7
RNA Integrity Number (RIN):	7 (B.02.08)
Result Flagging Color:	
Result Flagging Label:	RIN:7

Fragment table for sample 8 : A2

Name	Start Size [nt]	End Size [nt]	Area	% of total Area
18S	1,637	2,103	25.9	7.8
28S	2,432	3,504	44.7	13.5

B2

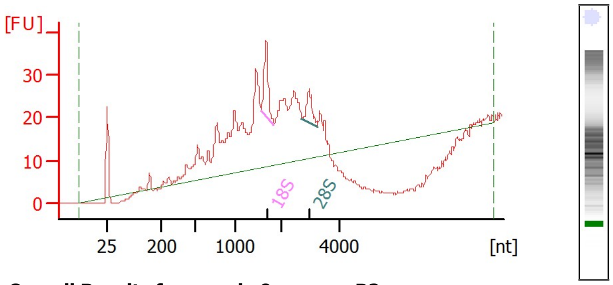

Overall Results for sample 9 : B2

RNA Area:	465.1
RNA Concentration:	148 ng/µl
rRNA Ratio [28s / 18s]:	0.5
RNA Integrity Number (RIN):	3.3 (B.02.08, Anomaly Threshold(s) manually adapted)
Result Flagging Color:	
Result Flagging Label:	RIN: 3.30

Fragment table for sample 9 : B2

Name	Start Size [nt]	End Size [nt]	Area	% of total Area
18S	1,567	1,842	24.0	5.2
28S	2,705	3,238	11.8	2.5

 © Copyright 2003 - 2009 Agilent Technologies, Inc.

Assay Class: Eukaryote Total RNA Nano
Data Path: \\1...Eukaryote Total RNA Nano_DE23101893_2015-05-06_09-36-22.xad
Created: 5/6/2015 9:36:22 AM
Modified: 5/6/2015 3:58:01 PM

Electropherogram Summary Continued ...

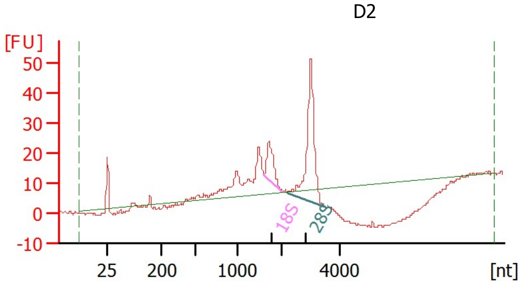

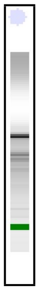

Overall Results for sample 10 : D2

RNA Area:	229.3
RNA Concentration:	73 ng/µl
rRNA Ratio [28s / 18s]:	3.8
RNA Integrity Number (RIN):	6.1 (B.02.08, Anomaly Threshold(s) manually adapted)
Result Flagging Color:	
Result Flagging Label:	RIN: 6.10

Fragment table for sample 10 : D2

Name	Start Size [nt]	End Size [nt]	Area	% of total Area
18S	1,605	1,972	26.6	11.6
28S	2,172	3,496	101.2	44.1

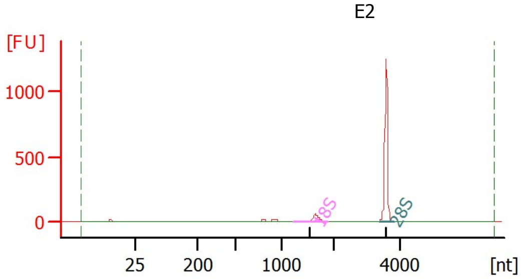

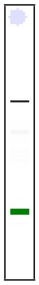

Overall Results for sample 11 : E2

RNA Area:	1,075.9
RNA Concentration:	341 ng/µl
rRNA Ratio [28s / 18s]:	7.8
RNA Integrity Number (RIN):	6.9 (B.02.08, Anomaly Threshold(s) manually adapted)
Result Flagging Color:	
Result Flagging Label:	RIN: 6.90

Fragment table for sample 11 : E2

Name	Start Size [nt]	End Size [nt]	Area	% of total Area
18S	1,202	1,878	99.6	9.3
28S	3,349	3,791	775.6	72.1

© Copyright 2003 - 2009 Agilent Technologies, Inc.

Assay Class: Eukaryote Total RNA Nano
Data Path: \\1...Eukaryote Total RNA Nano_DE23101893_2015-05-06_09-36-22.xad
Created: 5/6/2015 9:36:22 AM
Modified: 5/6/2015 3:58:01 PM

Electropherogram Summary Continued ...

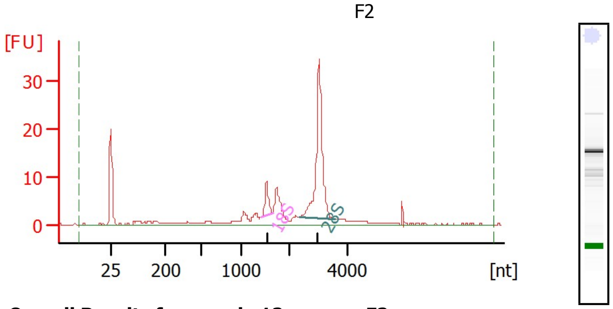

Overall Results for sample 12 : F2

RNA Area:	154.3
RNA Concentration:	49 ng/µl
rRNA Ratio [28s / 18s]:	6.7
RNA Integrity Number (RIN):	8.3 (B.02.08, Anomaly Threshold(s) manually adapted)
Result Flagging Color:	
Result Flagging Label:	RIN: 8.30

Fragment table for sample 12 : F2

Name	Start Size [nt]	End Size [nt]	Area	% of total Area
18S	1,438	1,654	8.6	5.6
28S	2,392	3,580	57.7	37.4

© Copyright 2003 - 2009 Agilent Technologies, Inc.
