# Supplementary material for: Gene Expression Analysis of Induced Plum pox virus (Sharka) Resistance in Peach (Prunus persica) by Almond (P. dulcis) Grafting
Source: Int J Mol Sci. 2021 Mar 30;22(7):3585. doi: 10.3390/ijms22073585 (PMC8036523; doi:10.3390/ijms22073585)
Supplement: Supplementary file 1 [file ijms-22-03585-s001.zip › ijms-1159338 R1 Supplementary Materials/ijms-1159338 R1 File S2.pdf]

Prupe.5G193400

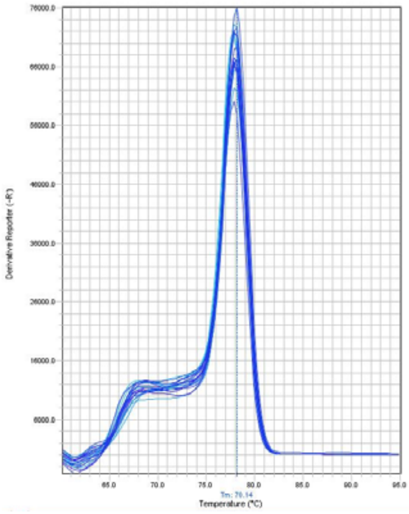

Prupe.1G453700

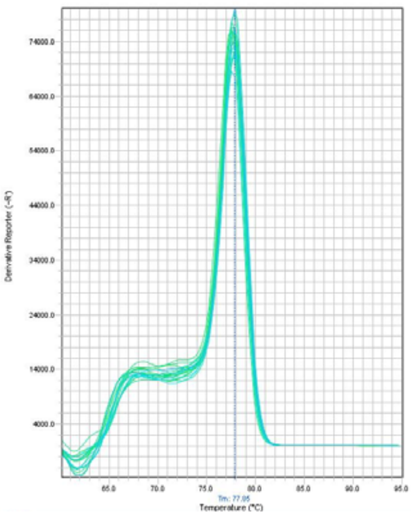

Prupe.7G194400

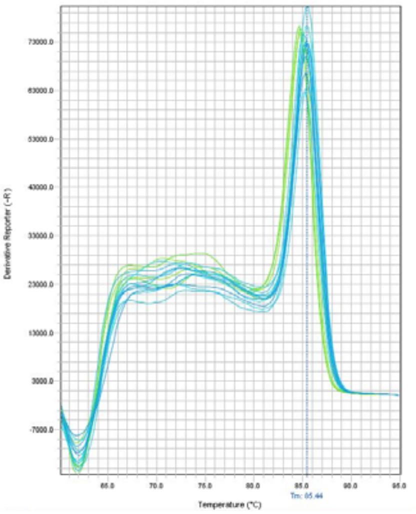

Prupe.4G176200

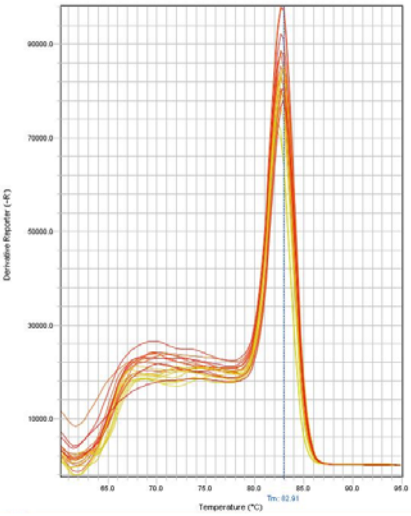

Prupe.1G393400

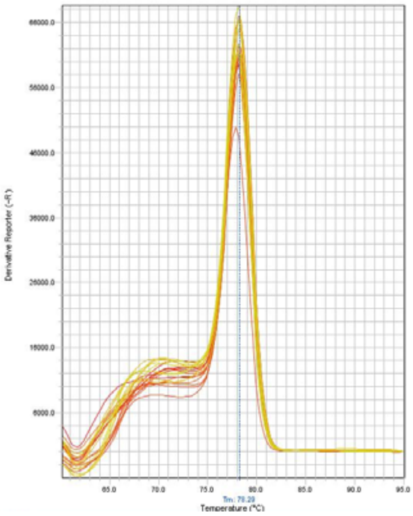

Prupe.5G164200

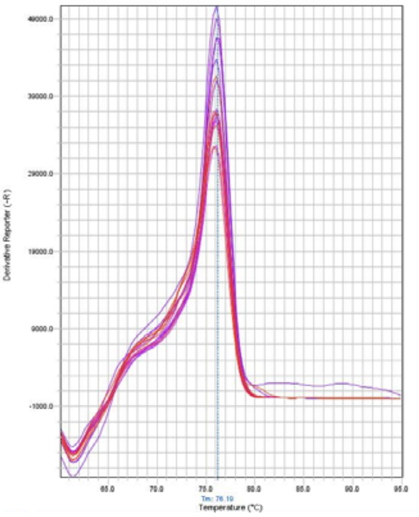

Melting temperatures for candidate genes involved in phytohormonal signalling.
Prupe.5G193400; *Gibberellin-regulated protein 4*
Prupe.1G453700; *Dehydration-responsive protein RD22*
Prupe.7G194400; *Ethylene-responsive transcription factor ERF017*
Prupe.4G176200; *Ethylene responsive element binding protein (ERBP)-like factor*)
Prupe.1G393400; *Chrorismate mutase*
Prupe.5G164200; *Cytochrome P450 71A1* (CYP71A1)

Prupe.8G163300

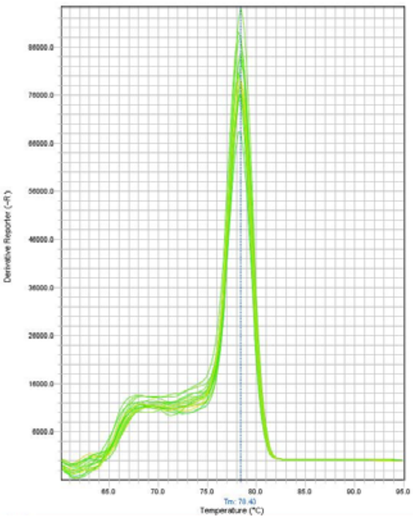

Prupe.1G039900

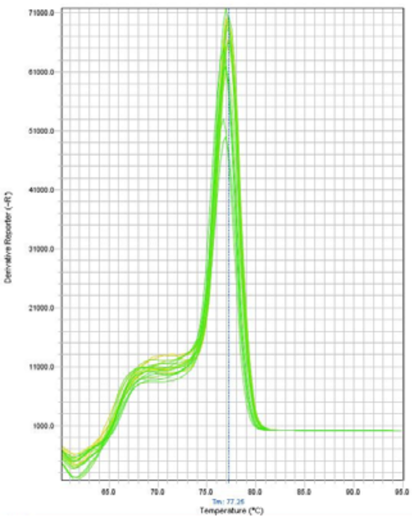

Prupe.1G091400

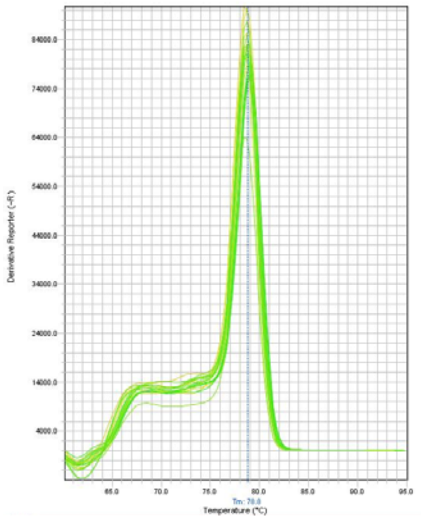

Prupe.2G204700

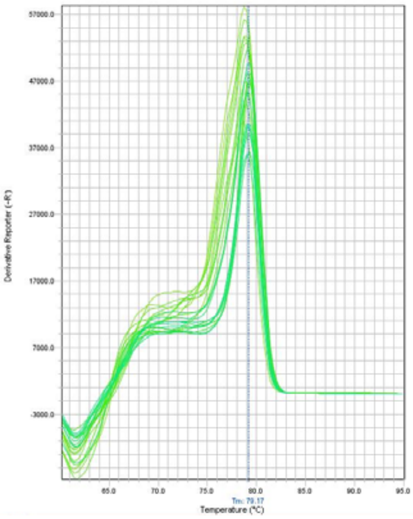

Prupe.3G255800

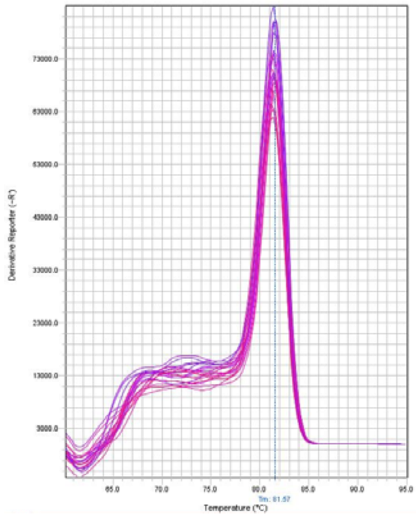

Prupe.7G051200

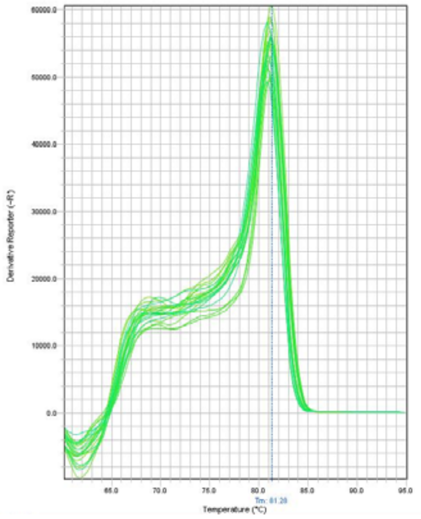

Prupe.7G048000

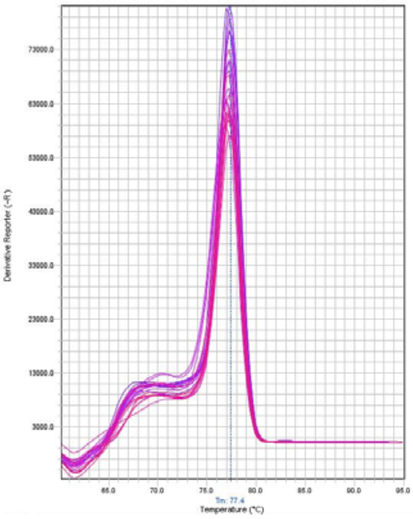

Prupe.1G488200

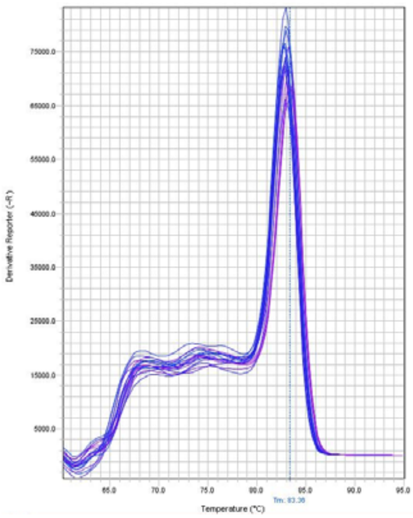

Melting temperatures for candidate genes related to pathogen response

Prupe.8G163300; *Pathogenesis-related thaumatin-like protein*
Prupe.1G039900; *Glutathione S-Transferase*
Prupe.1G091400; *Pathogenesis-related 1 protein*
Prupe.2G204700; *No apical meristem protein*
Prupe.3G255800; *Transcription initiation factor TFIIB*
Prupe.7G051200; *Glucan endo-1,3-beta-D-glucosidase*
Prupe.7G048000; *Endoribonuclease DICER homolog 2* (DCL2)
Prupe.1G488200; *Speckle-type POZ protein*

Prupe.2G263600

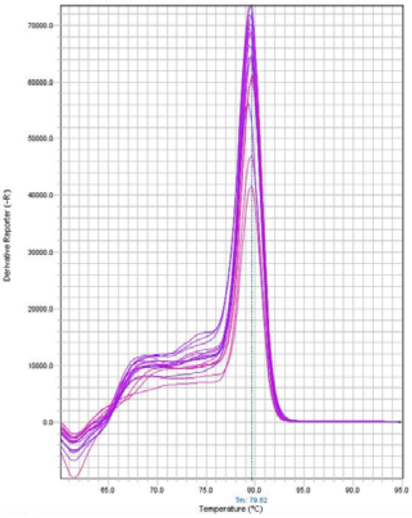

Prupe.8G132000

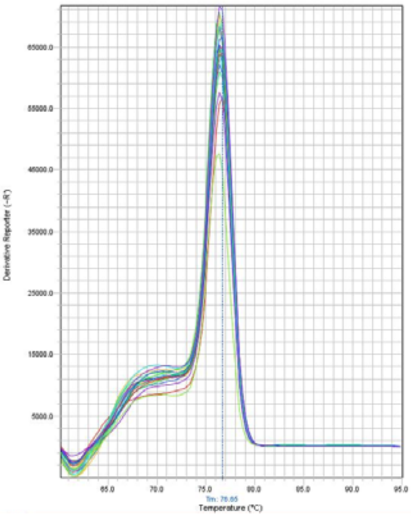

Prupe.6G163400

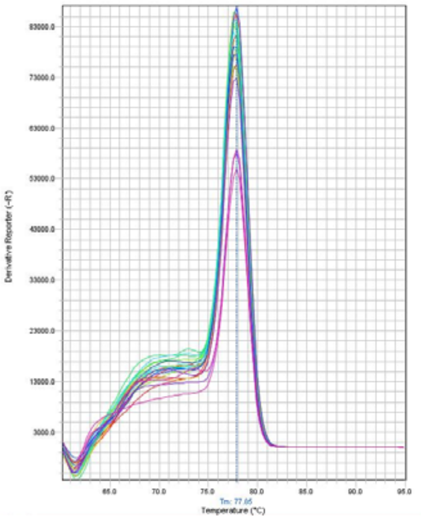

Melting temperatures for reference genes assayed
Prupe.2G263600; *Expansine-A8*
Prupe.8G132000; *RNA polymerase II*
Prupe.6G163400; *Actin*
